# Enabling Just-in-Time Clinical Oncology Analysis With Large Language Models: Feasibility and Validation Study Using Unstructured Synthetic Data

**DOI:** 10.2196/78332

**Published:** 2025-12-01

**Authors:** Peter May, Julian Greß, Christoph Seidel, Sebastian Sommer, Markus K Schuler, Sina Nokodian, Florian Schröder, Johannes Jung

**Affiliations:** 1 Department of Internal Medicine III, School of Medicine and Health, TUM University Hospital, Technical University of Munich, Ismaninger Str. 22, Munich, Germany, 49 89-4140-8753; 2 MPiriQ Science Technologies GmbH, Munich, Germany; 3 Department of Oncology, Hematology and Bone Marrow Transplantation with Division of Pneumology, University Medical Center Hamburg-Eppendorf, Hamburg, Germany; 4 MVZ Elisenhof, Munich, Germany; 5 Onkologischer Schwerpunkt am Oskar-Helene Heim, Berlin, Germany; 6 Department of Hematology and Medical Oncology, University Medical Center Göttingen, Göttingen, Germany

**Keywords:** large language models, clinical oncology, unstructured data, real-world evidence, natural language processing, survival analysis, cancer registries, just-in-time analysis, artificial intelligence

## Abstract

**Background:**

Traditional cancer registries, limited by labor-intensive manual data abstraction and rigid, predefined schemas, often hinder timely and comprehensive oncology research. While large language models (LLMs) have shown promise in automating data extraction, their potential to perform direct, just-in-time (JIT) analysis on unstructured clinical narratives—potentially bypassing intermediate structured databases for many analytical tasks—remains largely unexplored.

**Objective:**

This study aimed to evaluate whether a state-of-the-art LLM (Gemini 2.5 Pro) can enable a JIT clinical oncology analysis paradigm by assessing its ability to (1) perform high-fidelity multiparameter data extraction, (2) answer complex clinical queries directly from raw text, (3) automate multistep survival analyses including executable code generation, and (4) generate novel, clinically plausible hypotheses from free-text documentation.

**Methods:**

A synthetic dataset of 240 unstructured clinical letters from patients with stage IV non–small cell lung cancer (NSCLC), embedding 14 predefined variables, was used. Gemini 2.5 Pro was evaluated on four core JIT capabilities. Performance was measured by using the following metrics: extraction accuracy (compared to human extraction of n=40 letters and across the full n=240 dataset); numerical deviation for direct question answering (n=40 to 240 letters, 5 questions); log-rank *P* value and Harrell concordance index for LLM-generated versus ground-truth Kaplan-Meier survival analyses (n=160 letters, overall survival and progression-free survival); and correct justification, novelty, and a qualitative evaluation of LLM-generated hypotheses (n=80 and n=160 letters).

**Results:**

For multiparameter extraction from 40 letters, the LLM achieved >99% average accuracy, comparable to human extraction, but in significantly less time (LLM: 3.7 min vs human: 133.8 min). Across the full 240-letter dataset, LLM multiparameter extraction maintained >98% accuracy for most variables. The LLM answered multiconditional clinical queries directly from raw text with a relative deviation rarely exceeding 1.5%, even with up to 240 letters. Crucially, it autonomously performed end-to-end survival analysis, generating text-to-R-code that produced Kaplan-Meier curves statistically indistinguishable from ground truth. Consistent performance was demonstrated on a small validation cohort of 80 synthetic acute myeloid leukemia reports. Stress testing on data with simulated imperfections revealed a key role of a human-in-the-loop to resolve AI-flagged ambiguities. Furthermore, the LLM generated several correctly justified, biologically plausible, and potentially novel hypotheses from datasets up to 80 letters.

**Conclusions:**

This feasibility study demonstrated that a frontier LLM (Gemini 2.5 Pro) can successfully perform high-fidelity data extraction, multiconditional querying, and automated survival analysis directly from unstructured text. These results provide a foundational proof of concept for the JIT clinical analysis approach. However, these findings are confined to synthetic patients, and rigorous validation on real-world clinical data is an essential next step before clinical implementation can be considered.

## Introduction

### The Need for Timely Real-World Evidence in Oncology

Clinical registries are foundational to oncology research, providing essential real-world evidence on treatment patterns, patient outcomes, and therapeutic effectiveness beyond the confines of clinical trials. However, the conventional approach to building these registries—relying heavily on manual data abstraction from patient records—faces significant challenges. This process is labor-intensive and costly and introduces substantial time lags between clinical events and data availability. For example, manual abstraction of cancer stage can lag diagnosis by up to 6 months, hindering timely analysis [1]. Furthermore, traditional registries are inherently constrained by predefined data schemas, often failing to capture the rich, nuanced clinical details embedded within unstructured text like physician notes, pathology reports, or radiology findings [1,2]. Critical information, including precise biomarker status, evolving metastatic patterns, treatment responses, and subsequent lines of therapy, is also frequently updated, rendering static registries potentially outdated or incomplete for contemporary research needs.

### Automating Data Access: From Extraction to “Just-in-Time” Analysis

Advances in natural language processing, particularly the advent of powerful transformer-based large language models (LLMs), offer a promising avenue to overcome these limitations. Recent studies demonstrate the proficiency of LLMs in accurately extracting key clinical end points, such as real-world progression-free survival (PFS), biomarkers, and TNM stage, directly from unstructured clinical text, thereby reducing manual curation efforts [1,3-11].

Building upon this progress, we propose and investigate the concept of a “just-in-time” (JIT) clinical analysis paradigm in oncology. This approach leverages the advanced capabilities of LLMs to directly query and analyze unstructured clinical documents on-demand, potentially bypassing the slow, laborious process of consolidating data into static, structured databases. The central hypothesis is that LLMs can serve as intelligent interfaces to raw clinical data repositories—such as collections of electronic health record notes—enabling dynamic extraction, synthesis, and analysis of key oncology end points precisely when needed.

The latest generation of LLMs are pivotal enablers for this concept, largely because their technical capabilities now align with the demands of large-scale clinical data analysis. Context windows have increased immensely; Gemini 2.5 Pro, for instance, can handle 1 million tokens concurrently—enough to ingest and reason over hundreds of patient records or complex reports at once. While previous approaches, including methods like retrieval-augmented generation (RAG), enabled large-scale data processing, their accuracy and efficiency were often inconsistent [12,13]. The large context windows, combined with their sophisticated understanding of context, ability to synthesize information across documents, and emergent reasoning capabilities, position frontier LLMs not merely as data extractors but as potential “automated curators” or analytical engines operating directly on narrative data [5,14,15]. This could allow researchers and clinicians to pose complex analytical questions (eg, “What is the PFS for epidermal growth factor receptor–mutant lung cancer patients treated with osimertinib who developed brain metastases?") directly to document collections, with the LLM dynamically interpreting the query, locating relevant information scattered across notes, performing necessary calculations (like survival time), and providing synthesized answers on demand [16].

### Simulating "Just-in-Time" Querying With Synthetic Data

However, systematically evaluating the proposed JIT paradigm on real-world data is currently infeasible due to significant regulatory barriers. Privacy laws such as the US Health Insurance Portability and Accountability Act (HIPAA) or the EU General Data Protection Regulation prohibit the use of cloud-based LLMs with patient data. Moreover, the terms of use for publicly available datasets like Medical Information Mart for Intensive Care (MIMIC)-IV also restrict their integration with these models. Therefore, to rigorously assess the capabilities in a controlled yet clinically relevant setting, this study employs a well-established simulation framework using a synthetic dataset of 240 patients with stage IV non–small cell lung cancer (NSCLC), embedding predefined clinical variables within mock narrative summaries [17]. NSCLC was selected for its clinical heterogeneity and complexity, providing a representative testbed for evaluating LLM performance on information retrieval and reasoning tasks central to the JIT concept. This synthetic dataset serves as a consistent, accessible proxy for a collection of patient records, enabling systematic assessment of a cloud-based LLM (Gemini 2.5 Pro) in a reproducible environment.

### Rationale and Objectives

The primary objective of this study is to evaluate whether a leading LLM possesses the fundamental capabilities required to enable JIT analysis of complex oncological information. Specifically, we assess the LLM’s performance across four key tasks simulating the core functions of such a system:

1. High-fidelity structured parameter extraction: Can the LLM accurately extract numerous predefined variables, including PFS and overall survival (OS) from unstructured text at scale?
2. Direct clinical question answering: Can the LLM correctly answer complex queries requiring data aggregation and synthesis directly from text, bypassing a structured database?
3. Automated survival analysis: Can the LLM perform multistep analyses, like generating Kaplan-Meier curves from raw text, yielding results comparable to traditional methods?
4. Autonomous hypothesis generation: Does the LLM exhibit reasoning capabilities by identifying potentially novel clinical associations?

While many studies have demonstrated the ability of LLMs to extract specific data points from clinical text, the primary contribution of our feasibility study is not algorithmic but paradigmatic. We propose that, in the future, the use of LLMs in cancer registries may obviate the need for intermediate structured databases.

## Methods

### Synthetic Patient Data Generation

To create a controlled benchmarking environment, a synthetic dataset simulating clinical correspondence from 240 patients with stage IV NSCLC was generated. A structured data schema defining 14 key clinical variables (including demographics, diagnosis, metastases, molecular markers, treatments, PFS, and OS; details in Multimedia Appendix 1) was created manually and served as the ground truth (GT). Unstructured patient letters embedding these variables were generated using GPT o4-mini and Claude Sonnet 3.7, mimicking real-world linguistic diversity (examples in Multimedia Appendix 2).

A key goal was to create a heterogeneous dataset to better simulate the variability of real-world clinical documentation. As detailed in Table S1 (Multimedia Appendix 2), the resulting 240 letters vary significantly in style, length, and granularity, with word counts ranging from 109 to 3147 words. The dataset includes extremely concise, 120-word Subjective, Objective, Assessment, and Plan (SOAP) notes rich in abbreviations (eg, SYN092, shown in Multimedia Appendix 2), alongside comprehensive, 3063-word discharge summaries containing detailed laboratory results, pathology reports, and extensive narrative histories (eg, SYN007, shown in Multimedia Appendix 2). For validation purposes, a second, smaller dataset with 80 patients with acute myeloid leukemia (AML) was created (Table S2 in Multimedia Appendix 2). Each synthetic letter underwent a basic plausibility assessment by an oncologist to ensure suitability for testing.

### Large Language Model Setup

All analytical tasks utilized Gemini 2.5 Pro accessed via Google AI Studio, prompted in a zero- or one-shot fashion with temperature set to 0 for deterministic output. Session history was disabled to ensure reproducibility. Distinct models for generation and analysis mitigated self-evaluation bias. Due to the platform’s limit on concurrent file uploads, the synthetic letters were batched into multipage PDF files, each containing 20‐40 patients. A detailed flowchart providing an overview of the entire experimental design is provided in Figure S1 (Multimedia Appendix 2).

### Parameter Extraction Performance

The LLM’s ability to extract the 14 predefined variables was evaluated in single parameter extraction (SPE; one variable at a time) and multiparameter extraction (MPE; all 14 variables simultaneously) modes. OS and PFS had to be calculated using the difference between progression and treatment initiation date in most cases. For initial benchmarking, human performance on a set of 40 letters was compared with LLM SPE and MPE performance (6 independent repetitions). Next, the impact of input volume (n=240 letters processed in batches of 1×240, 2×120, 3×80, 4×60, or 6×40) was assessed. Accuracy (ratio of correct predictions) and processing time were recorded. A ±1 month tolerance was applied for OS/PFS time values.

### Question Answering Accuracy and Deviation

The LLM’s capacity for direct question answering was tested using subsets of the patient letters, ranging from 40 to 240 letters in increments. Five questions of increasing complexity were posed: Q1 (1 parameter): How many patients have brain metastasis at diagnosis? Q2 (1 parameter): How many patients were diagnosed in 2020? Q3 (1 parameter): How many patients received tyrosine kinase inhibitors for first-line therapy? Q4 (2 parameters): How many female patients have epidermal growth factor receptor mutations? Q5 (3 parameters): How many patients born before 1970 have a KRAS mutation and are on Pembrolizumab-containing first-line regimens? Each question was tested with different quantities of letters (40-240) across 12 independent replicates to assess consistency. Evaluation metrics included the percentage of correct replicates (the proportion of the 12 replicates where the LLM provided the exactly correct numerical answer based on the GT data for the given subset of letters) and relative deviation (absolute difference between the LLM’s answer and the true answer, divided by the quantity of letters used for that query).

### Automated Survival Analysis

We assessed the LLM’s ability to perform an end-to-end survival analysis using a cohort of 160 letters. The LLM was prompted to extract time-to-event data (for OS and PFS) by generating a downloadable table and a complete, executable R script using the survival and survminer packages to generate Kaplan-Meier plots. This process was also applied to a more complex subgroup PFS analysis, stratifying patients by driver mutation status (driver mutation versus wild type [WT]). This semiautomated workflow required a brief human-in-the-loop action: the user had to save the LLM-generated data table as a CSV file and then execute the LLM-generated script in RStudio. This manual step reflected a current platform limitation: although Gemini 2.5 Pro can execute Python to generate Kaplan-Meier curves, it cannot do so while simultaneously extracting data from uploaded PDF files. The detailed prompt and a complete sample of the LLM’s output (data table and R code) are provided in A2.7 and A2.8 in Multimedia Appendix 2. The resulting curves were compared visually and statistically (log-rank test) against GT data. To assess reproducibility, Harrell’s concordance index (C-index) was calculated for 12 independent repetitions, where a value of 0.50 signified that the survival curves derived from the LLM and the GT were statistically indistinguishable.

### Error Injection Stress Test

To simulate real-world data imperfections, we conducted a stress test on a subset of 40 letters, each intentionally altered to include one typographical error, one data ambiguity/contradiction, and one missing data point (see Table S3 in Multimedia Appendix 2 for injected errors). We compared two conditions in 3 independent repetitions: (1) “LLM MPE only,” where the LLM performed direct MPE on the error-injected text, and (2) “Human-in-the-loop,” a two-stage workflow where the LLM first flagged potential errors for a human annotator to review (see prompt and exemplary output in A2.9 and A2.10 in Multimedia Appendix 2). For each LLM-flagged error, the human annotator added the clarification to a subsequent MPE prompt, with which the final parameter extraction was performed. The number of correctly extracted parameters (of all 40 corrupted data points) was recorded for each condition.

### Autonomous Hypothesis Generation

To evaluate the LLM’s reasoning capabilities, we prompted it to analyze the full dataset of unstructured letters (in batches of n=80 and n=160) and propose a single, novel, and clinically relevant hypothesis regarding potential relationships within the cohort. The prompt was designed to elicit one focused hypothesis per run to ensure high-quality output (prompt in A2.12 in Multimedia Appendix 2). Each generated hypothesis subsequently underwent a structured, qualitative evaluation by a board-certified oncologist. The evaluation was based on three distinct criteria:

- Justification: Assessed whether the justification for the hypothesis was factually supported by the provided synthetic data. This was rated as “fully correct,” “partly correct,” or “incorrect.”
- Biological plausibility: Assessed the clinical and biological sensibility of the proposed association, rated as “high,” “moderate,” or “low.”
- Novelty: Assessed whether the hypothesis represented a nonobvious relationship not considered standard clinical knowledge, rated as “high,” “moderate,” or “low.”

The proposed hypotheses and the detailed expert evaluation are provided in Table S4 (Multimedia Appendix 2).

### Statistical Analysis and Visualization

Performance metrics, including accuracy, processing time, and relative deviation, were summarized using descriptive statistics (mean, standard error of the mean, and range). Kaplan-Meier curves were generated using the R survival and survminer packages. Log-rank tests and Harrell’s C-index were used to compare survival distributions. Data visualization was performed using GraphPad Prism (version 10.4.2; GraphPad Software, LLC) and R (version 4.5.0; R Core Team, 2025) with packages ggplot2 and caret. Conceptual visualizations were prepared using BioRender.

### Ethical Considerations

This study utilized exclusively synthetic data generated by LLMs. No real patient data or protected health information was accessed or used at any stage. As only synthetic data were used, this study did not involve human subject research requiring institutional review board approval or informed consent. The TRIPOD-LLM reporting guideline for studies using LLMs was completed.

## Results

### High-Fidelity Information Extraction From Unstructured Clinical Texts

First, we benchmarked the LLM’s ability to extract structured clinical variables against manual human annotation. On a 40-letter synthetic dataset with 14 parameters per patient, both LLM SPE and MPE achieved >99% average accuracy (SPE: 99.8%; MPE 99.5%). As shown in Table 1, the results were comparable to those of a human annotator (98%). Critically, the LLM MPE approach demonstrated superior time efficiency, processing the 40-letter batch in 3.7 minutes, compared to 46.3 minutes for LLM SPE and 133.8 minutes for manual extraction. In other words, the LLM performed the task with slightly higher accuracy in less than 3% of the time compared to human annotators.

**Table 1.** Benchmarking information extraction accuracy and processing time.

Extraction method	Accuracy (%)	Range (%)	Time taken (min)	Range (min)
Human	98.0	—^a^	133.8	—
LLM^b^ SPE^c^^,d^	99.8	99.1‐100.0	46.3	42.5‐51.4
LLM MPE^d^^,^^e^	99.5	99.1‐99.8	3.7	3.3‐4.2

a Not applicable.

b LLM: large language model.

c SPE: single parameter extraction.

d LLM approaches were tested on 6 different sets of 40 letters.

e MPE: multiparameter extraction.

### Effective Scaling to Larger Batch Sizes

**Figure 1.**
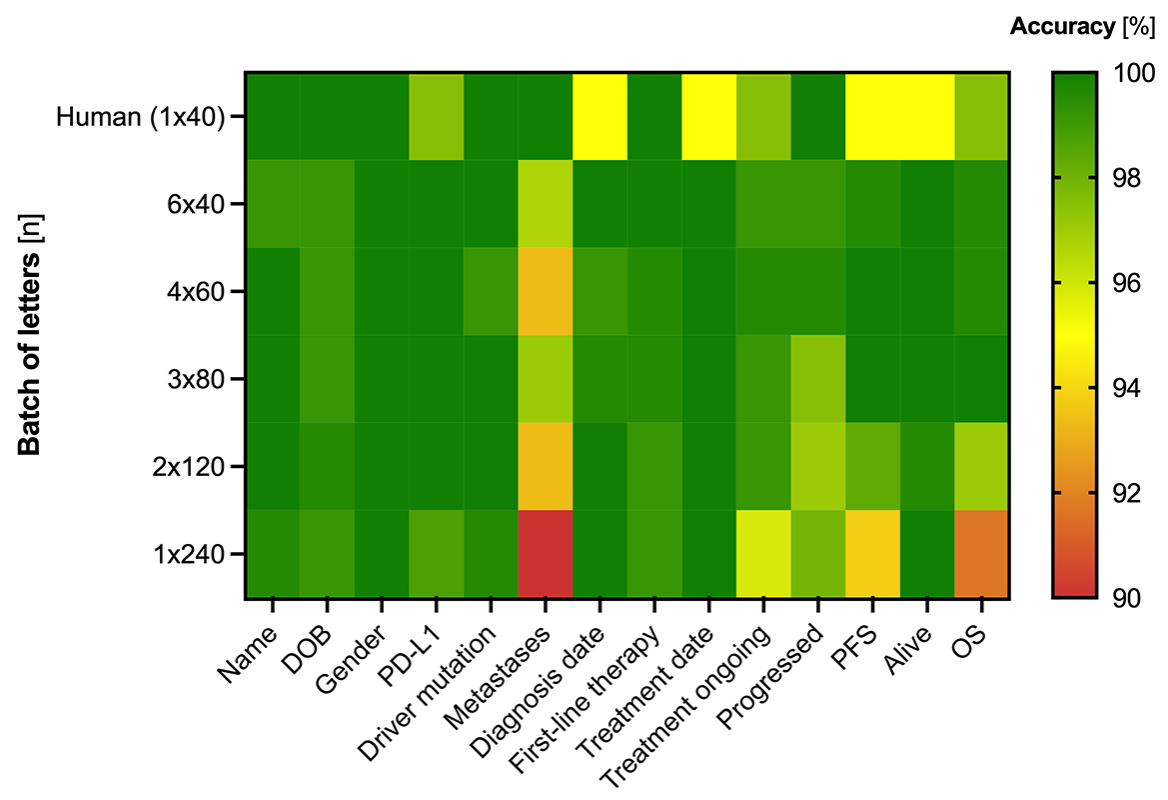
Extraction accuracy across clinical variables and batching strategies. The heatmap visualizes the accuracy across 14 clinical variables (x-axis) when processing the full 240-letter cohort, using five different batching strategies (y-axis), with a human annotator’s performance on a 40-letter batch included for reference. The color of each cell corresponds to the accuracy percentage from high (green, 100%) to low (red, 90%). DOB: date of birth; PD-L1: programmed death-ligand 1; PFS: progression-free survival; OS: overall survival.

The LLM MPE method scaled effectively to the full 240-letter dataset. Confusion matrices for “Alive status” and “Progression status” (1×240 batch) confirmed high classification accuracy of almost 100% (Figure S2 in Multimedia Appendix 2). Across all parameters and various batching strategies, LLM MPE generally maintained >98% accuracy (Figure 1). While errors were slightly higher for complex variables (eg, PFS, OS, metastases), they rarely exceeded 5%. A qualitative analysis revealed that a primary source of these errors was the model’s occasional misinterpretation of inconsistent date formats. Heatmap visualizations revealed that smaller batch sizes (eg, 6×40) modestly but consistently improved accuracy for some variables, suggesting potential advantages of minimizing input volume per prompt.

The overall processing time for the 240 letters using different batching strategies ranged from 15 to 30 minutes (Figure S3A, B in Multimedia Appendix 2), with MPE offering marked per-parameter efficiency over SPE. Although less time efficient, the SPE approach generally achieved slightly higher accuracies across all categories than MPE (Figure S3C in Multimedia Appendix 2).

These results indicate that LLM-driven MPE is a highly accurate and exceptionally time-efficient method for extracting structured data from clinical narratives, providing a robust foundation for subsequent complex analyses.

### Question-Answering Performance Across Variable Text Lengths

While the successful extraction of large volumes of structured data (up to 14×240=3360 parameters) highlights the LLM’s potential as a high-throughput database generator, we next asked a more fundamental question: is a structured database even necessary? To explore this, we evaluated whether the LLM could bypass database construction entirely and instead directly answer clinical queries from raw, unstructured patient letters across varying input sizes (n=40 to 240). Five questions were posed, requiring aggregation of one (Q1–3), two (Q4), or three (Q5) parameters simultaneously.

**Figure 2.**
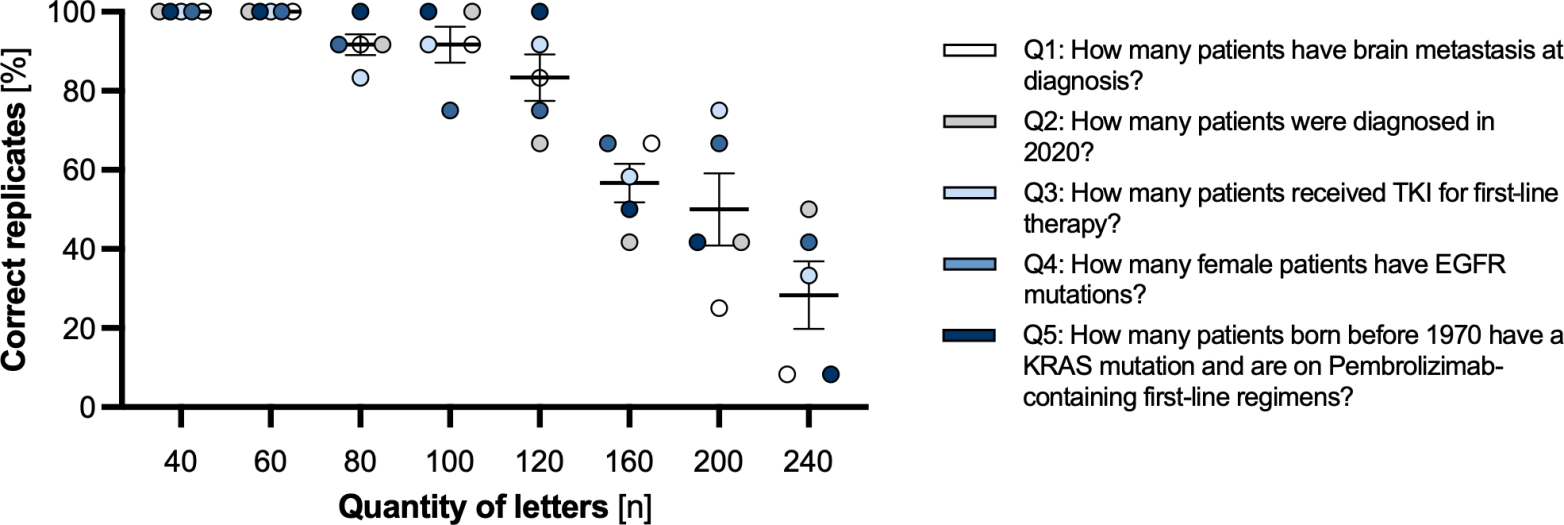
Percentage of correct replicates in direct question answering from unstructured text with increasing input. Performance was assessed using varying quantities of text from patient letters (40‐240 letters) and five questions (Q1–Q5) regarding 1 (Q1–3), 2 (Q4), or 3 (Q5) parameters (defined in legend). 12 replicates per condition. Error bars represent standard error of the mean. EGFR: epidermal growth factor receptor; KRAS: Kirsten rat sarcoma viral oncogene homolog; TKI: tyrosine kinase inhibitor.

Accuracy was highest when the LLM processed smaller text volumes. Performance was excellent with 40 or 60 letters and remained strong with 80 or 100 letters, particularly for simpler single-parameter questions (Q1–3; Figure 2). Notably, accuracy in obtaining the *exact* count decreased as the input volume increased further toward 240 letters, especially for the multiparameter questions (Q4–5).

**Figure 3.**
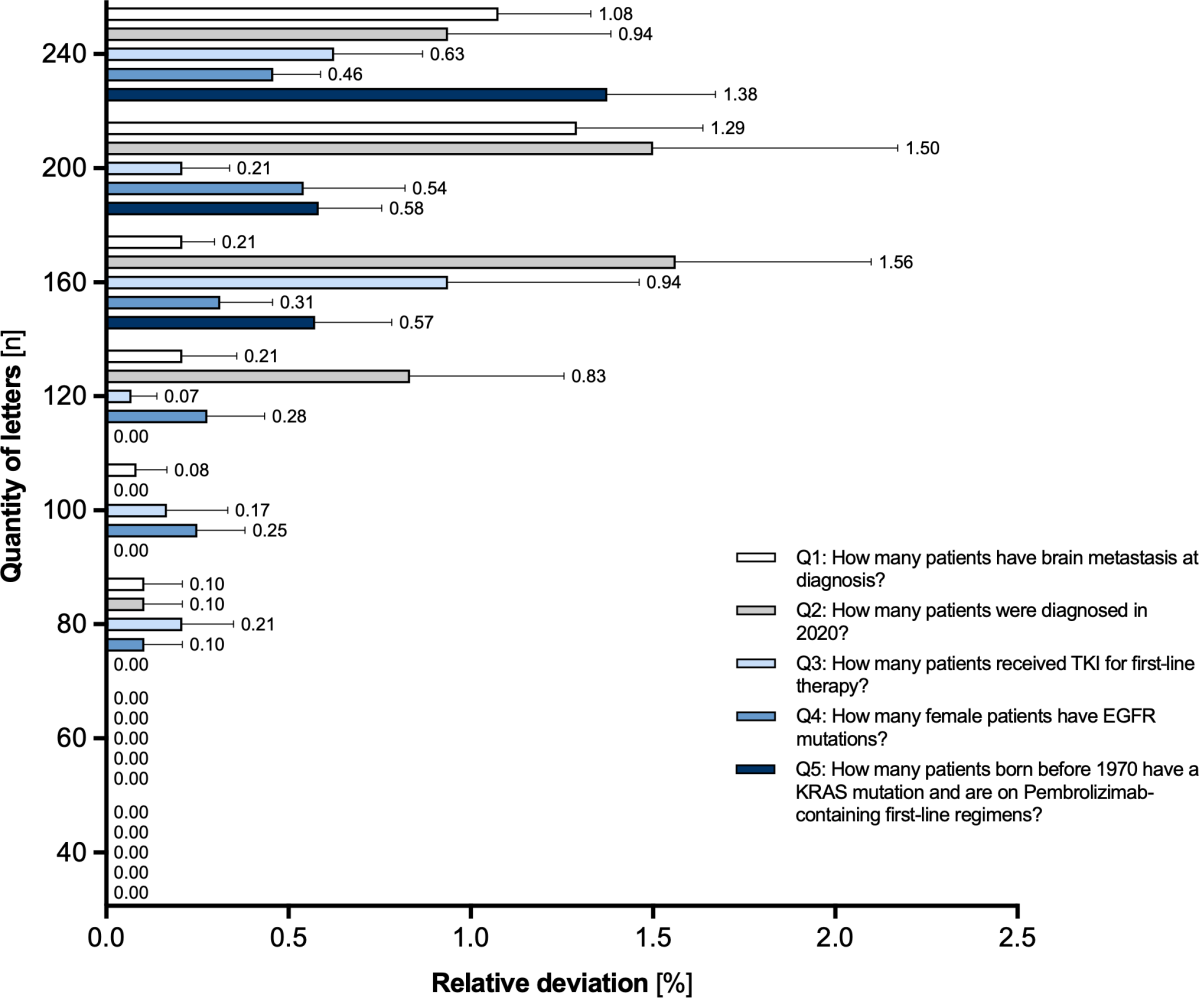
Relative deviation in direct question answering from unstructured text with increasing input. Relative deviation was measured as absolute deviation divided by correct value. Error bars represent standard error of the mean. EGFR: epidermal growth factor receptor; KRAS: Kirsten rat sarcoma viral oncogene homolog; TKI: tyrosine kinase inhibitor.

However, the answers remained precise in relative terms. Normalized by the correct answer, the magnitude of the errors was consistently small, typically remaining below 1% across most conditions and rarely exceeding 1.5% (Figure 3). Furthermore, there was no significant difference in the relative error magnitude between the simpler single-parameter questions (Q1–3) and the more complex multiparameter questions (Q4–5). This robustness supports the feasibility of using LLMs for large-scale JIT interrogation, where approximate accuracy within a small margin is often sufficient. It also underscores the potential of bypassing traditional structured databases altogether.

### Automated Survival Analysis Yields High Concordance With Ground Truth

Although direct question answering demonstrated strong performance and low deviation even across large text inputs, many clinical tasks demand more complex, multistep reasoning. Survival analysis, in particular, requires identifying event timing, censoring, and proper cohort stratification—tasks traditionally dependent on curated datasets and statistical programming. We therefore assessed whether the LLM could autonomously extract time-to-event data from unstructured patient letters (n=160) and generate fully executable R code to produce survival curves comparable to those derived from GT data.

**Figure 4.**
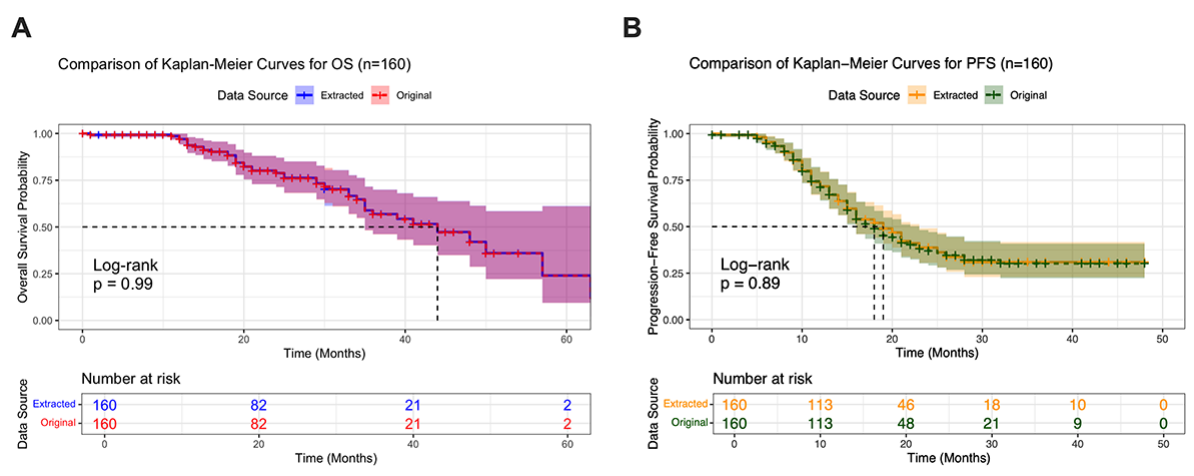
Direct unstructured text-to-R code Kaplan-Meier curves. Comparison of “extracted” versus ground truth (“Original”) for (A) overall survival (OS) and (B) progression-free survival (PFS) using 160 patients. Shaded areas represent 95% CI. Numbers-at-risk tables are shown below each plot. Log-rank tests assessed differences between the extracted and original curves.

Visual comparison of survival curves generated from LLM-extracted versus GT datasets revealed remarkable concordance, with numbers-at-risk tables almost identical. For both OS (Figure 4A) and PFS (Figure 4B), the shapes of survival curves were virtually indistinguishable across datasets, with overlapping 95% CI. Log-rank tests confirmed this observation, yielding *P* values indicating no statistically significant difference between the LLM-extracted and original datasets (*P*=.99 for OS; *P*=.89 for PFS).

**Figure 5.**
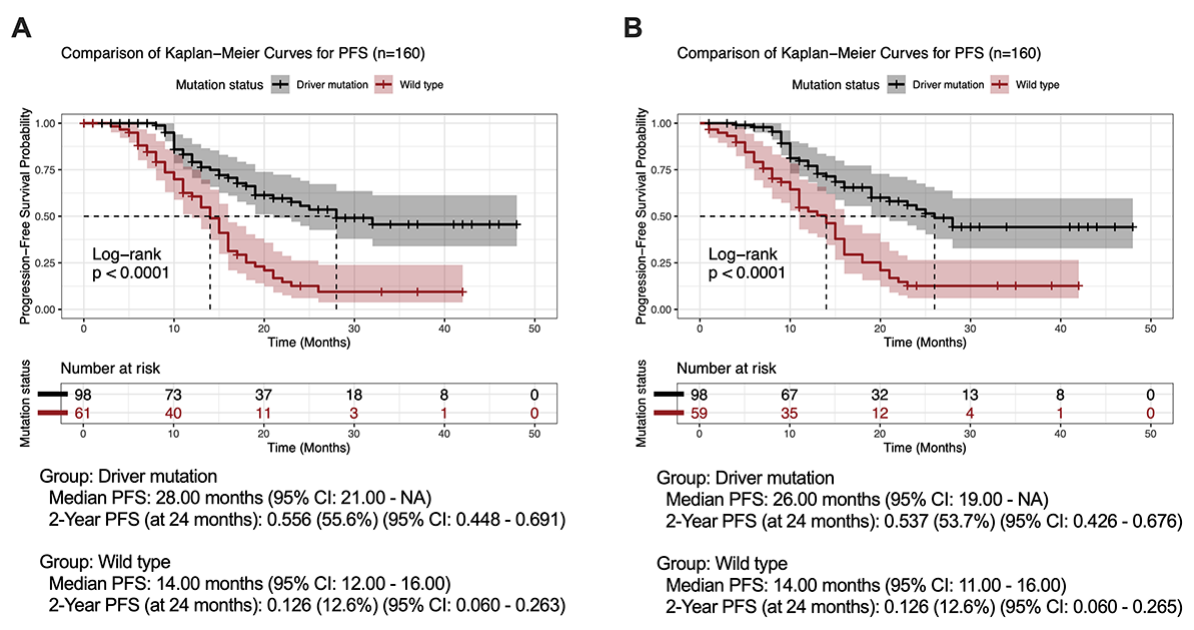
Direct unstructured text-to-R code subgroup analyses. Subgroup progression-free survival (PFS) analysis using the original data (A) and performed by the large language model (B) using data from 160 letters, comparing driver mutation (black) versus wild type (brown). Shaded areas represent 95% CI. Numbers-at-risk tables are shown below each plot. Log-rank *P* values test for differences between groups. Median and 2-year PFS estimates are provided.

Moreover, subgroup survival analyses were successfully performed. When stratifying patients by driver mutation status using data extracted from 160 letters, the LLM was able to reproduce prognostic distinctions (Figure 5A and B), generating distinct survival curves for driver mutation versus WT cohorts. It accurately calculated relevant statistics with only slight deviations for the driver mutation group, yielding comparable median PFS (26.0 instead of 28.0 mo for driver mutation versus 14.0 mo for WT) and 2-year PFS estimates (53.7 instead of 55.6% for driver mutation versus 12.6% for WT). The LLM also correctly identified the highly significant difference between these molecularly defined subgroups via log-rank testing (*P*<.001).

To confirm the robustness and reproducibility of the survival analysis, we repeated the semiautomated process 12 independent times (Figure S4 in Multimedia Appendix 2). The resulting survival models showed high stability, with Harrell’s C-index consistently ranging from 0.493 to 0.521, indicating that the LLM-extracted curves were statistically indistinguishable from the GT (C-index=0.500).

### Validation on an Independent Leukemia Cohort

Next, to assess the generalizability of our approach, we conducted a validation study on a second, independent synthetic cohort. This dataset comprised 80 patients with AML, a hematological malignancy with distinct clinical terminology and data structures (Table S2 in Multimedia Appendix 2). We repeated the core analytical tasks, including MPE, direct question answering, and automated survival analysis. The LLM’s performance on the AML dataset was highly consistent with the NSCLC cohort, achieving an overall MPE accuracy of 99.7% on batches of 40 letters, answering complex questions with a relative deviation below 0.5%, and generating survival curves nearly identical to GT (Figure S5 in Multimedia Appendix 2).

### Stress Testing the Framework Against Simulated Real-World Data Imperfections

**Figure 6.**
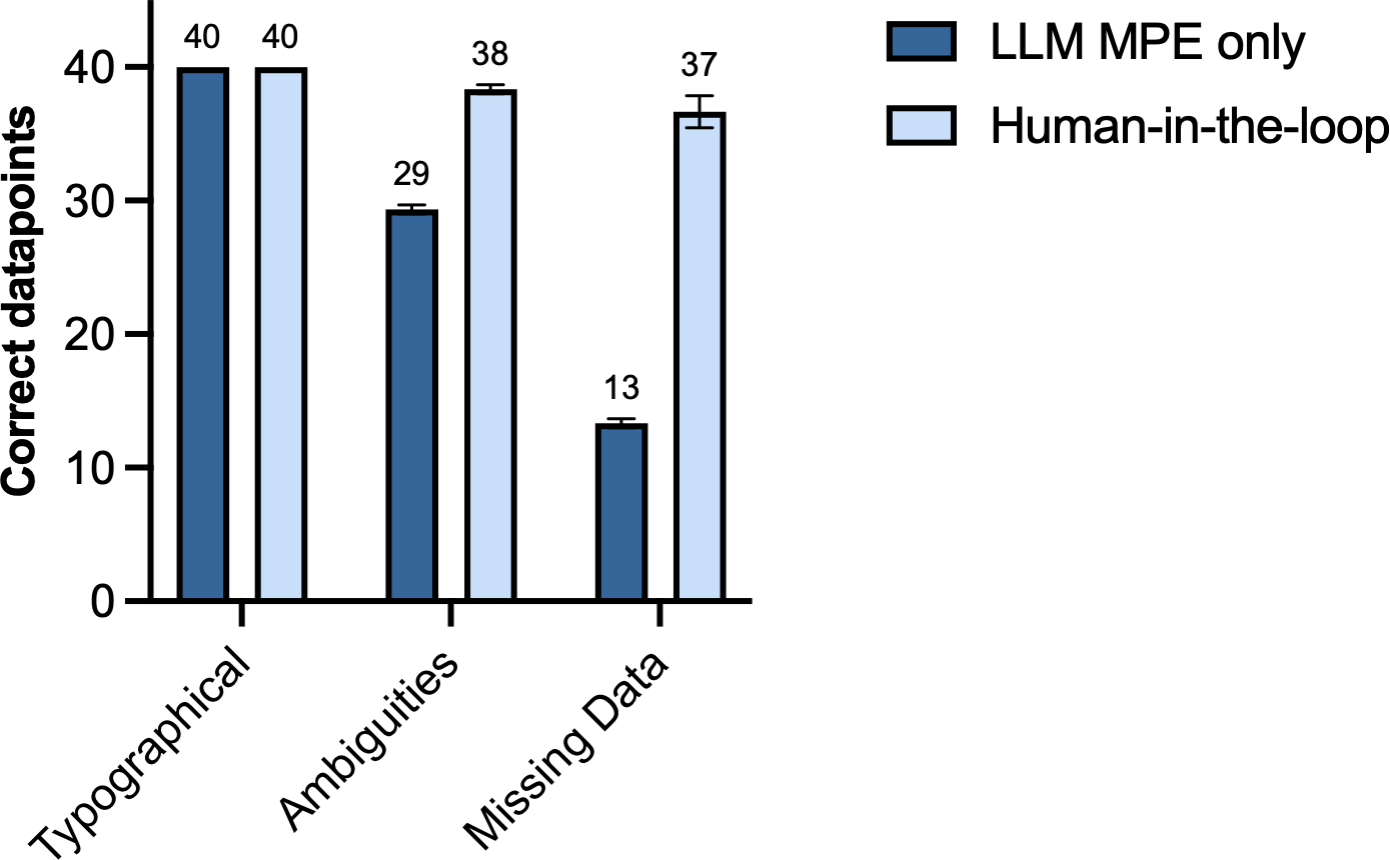
Effect of data quality issues. Bar chart comparing direct LLM extraction (“LLM MPE only”) against a “human-in-the-loop workflow” on a dataset corrupted with 40 typographical errors, ambiguities, and missing data. Bars show the mean correct data points (out of 40) from 3 independent repetitions; error bars represent the standard error of the mean. LLM: large language model; MPE: multiparameter extraction.

Given the limitations of synthetic data, we sought to assess the framework’s resilience to common real-world data challenges. We therefore conducted a stress test on a modified subset of 40 letters, where each letter was intentionally corrupted with one relevant typographical error, one data ambiguity or contradiction, and one missing data point. In the MPE task, the LLM demonstrated high resilience to typographical errors, with no impact on accuracy. However, data ambiguities, contradictions, and especially missing data substantially reduced performance (Figure 6). To address this, we evaluated a two-stage workflow: errors or missing data were first flagged by the LLM and then resolved by a human annotator. With the human-in-the-loop approach, almost all deliberately introduced ambiguities and missing data points were resolved.

### Hypothesis Generation Beyond Extraction

Having established the LLM’s ability to replicate conventional analytic workflows, we turned to a more exploratory objective: could the model go beyond predefined tasks and autonomously generate novel, clinically meaningful hypotheses? To test this, we prompted the LLM to analyze the dataset (n=80 and n=160 letters) and independently propose potential associations or insights. One example for such a hypothesis (H1) was: “In patients with KRAS-mutated NSCLC treated with an immune checkpoint inhibitor-containing regimen, the presence of a concurrent serine/threonine kinase 11 loss-of-function mutation is associated with a significantly increased risk of developing severe (Grade 3) immune-mediated colitis.”

As shown in Table 2, with 80 letters, the LLM offered entirely correct justifications from the provided data. However, with 160 letters, performance dropped and most explanations were either only partly correct or incorrect. Moreover, biological plausibility was generally moderate to high, while the novelty of the offered hypotheses tended to be only moderate.

**Table 2.** Evaluation of hypothesis generation capabilities.

Hypothesis	Justification	Biological plausibility	Novelty
80 letters			
H1	Fully correct	Low-moderate	High
H2	Fully correct	Moderate	Moderate-high
H3	Fully correct	Moderate-high	Moderate
H4	Fully correct	High	Low-moderate
H5	Fully correct	High	Low-moderate
160 letters			
H6	Partly correct	Low-moderate	High
H7	Incorrect	High	Low-moderate
H8	Fully correct	High	Low
H9	Partly correct	High	Moderate
H10	Incorrect	High	Moderate

## Discussion

### Principal Results

This study provides proof-of-concept that LLMs can significantly advance the processing of unstructured clinical oncology data. Our results demonstrate the feasibility of this approach: we show that LLMs can not only reliably and efficiently extract structured parameters but also perform complex multistep analyses like Kaplan-Meier survival estimation. A stress test designed to simulate real-world ambiguities, noise, and missing data showed that while performance degrades under these conditions, this degradation can be effectively mitigated using a human-in-the-loop approach. Finally, for smaller batches, biologically plausible hypotheses were generated from unstructured data without any prior human annotations or prompts about specific associations. While this study did not formally test the proposed hypotheses, their justification from the data source and novelty reinforce the potential for LLMs to function not only as passive extractors but also as proactive scientific agents within the clinical workflow.

**Figure 7.**
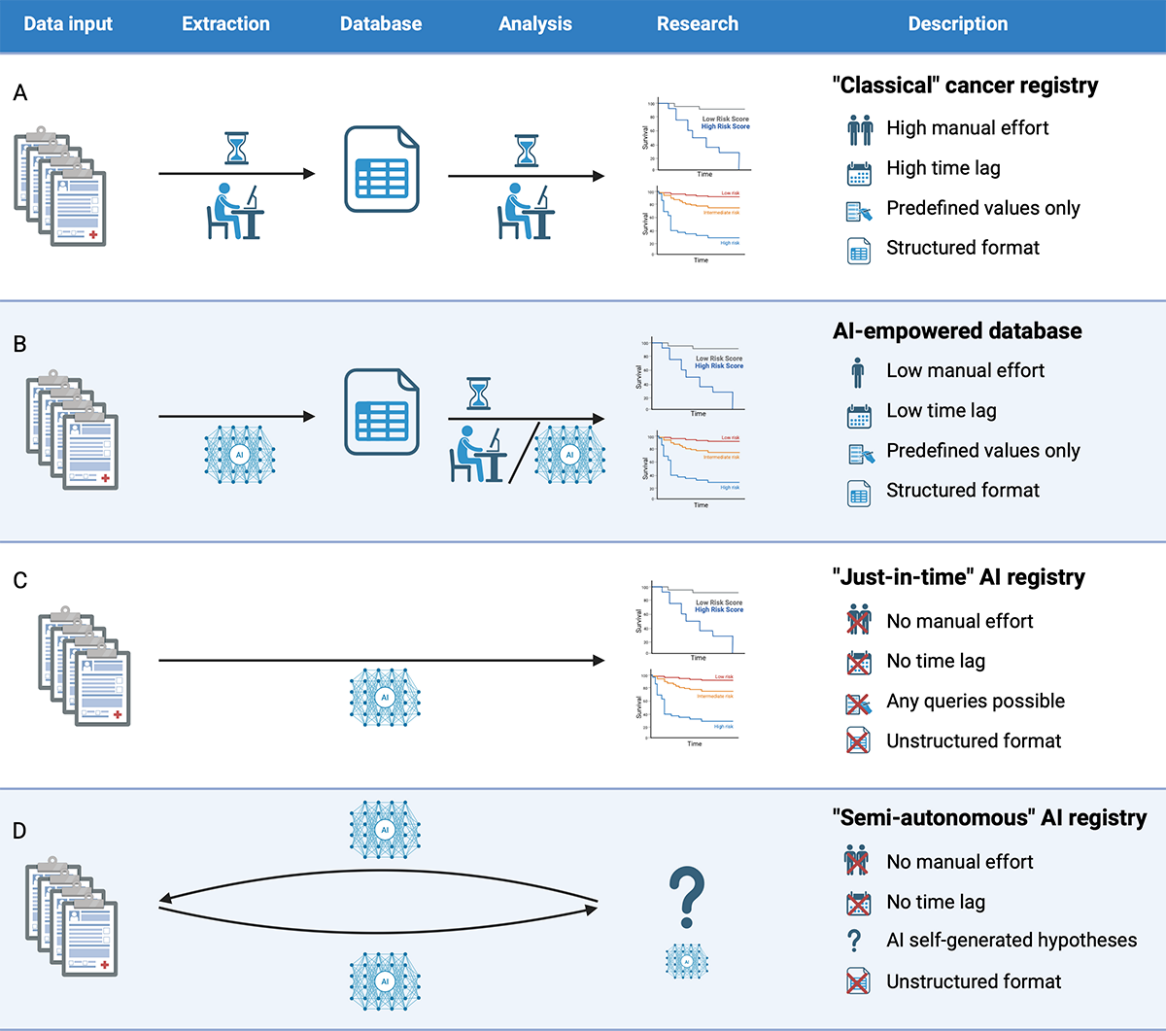
Schematic overview of different cancer registry paradigms. Comparison of workflow and characteristics for (A) classical manual registry, (B) AI-empowered extraction feeding a structured database, (C) direct AI analysis of unstructured data (“Just-in-time”), and (D) a future concept of a semiautonomous, hypothesis-generating AI registry. AI: artificial intelligence.

As conceptualized in Figure 7, these findings support a potential paradigm shift for oncology data management in the future: from static, delay-prone databases toward dynamic, JIT analysis of raw clinical documentation [14,15], potentially accelerating research [1,2,18].

While prior work has demonstrated LLM proficiency in extracting specific clinical end points like PFS [3,4], biomarkers [5], and TNM stage [1] from unstructured text, often focusing on single-task extraction to populate structured databases, our study extends these findings in several key dimensions. The novel contribution of our work is the systematic assessment of an LLM as an integrated interface for complex, multistep tasks in clinical oncology research, potentially obviating the need for intermediate structured databases for many analytical needs. In contrast to the limitations of manual methods or static natural language processing approaches, LLMs exhibit flexible reasoning, adapt to narrative variability, and synthesize context across documents [3,5,19,20]. Our work quantifies this fidelity at scale, showing LLMs maintain clinically acceptable error rates in high throughput, with multiple complex parameters extracted simultaneously. In addition to speed, LLMs offer crucial flexibility: new parameters can be readily incorporated, allowing analytical frameworks to evolve with scientific understanding—a significant advantage over static traditional registries.

However, the practical feasibility of a JIT approach hinges on specific economic and privacy considerations. Regarding cost, we estimated the computational expense on a single analytical run on our full 240-letter cohort at current application programming interface (API) pricing to be around US $0.30–$0.40. In line with our human benchmark, prior studies have reported 225 person-hours for manual abstraction of 100 complete patient records, making estimated labor costs for such tasks much higher [21]. Whether it is more economical to use LLMs to perform a one-time MPE to create a structured database, with subsequent analyses run on that database, or always interrogate the entire batch of unstructured data, will depend on future API pricing. Beyond cost, translating the JIT approach to real patient data necessitates strict attention to privacy and regulations like HIPAA or General Data Protection Regulation [22]. A primary pathway involves using secure, HIPAA-compliant cloud APIs from major providers, which are of state-of-the-art performance but rely on the provider’s security architecture. An alternative is the deployment of local, on-premise LLMs, which provide maximum data security and control but currently lag behind frontier models in analytical capability and require significant institutional investments in hardware and maintenance [23,24]. Therefore, a successful real-world implementation must carefully balance the analytical power of cloud models with the security of local solutions, potentially through hybrid approaches or advanced, LLM-based deidentification techniques [25].

### Limitations

Our study has several limitations that must be considered when interpreting the results. First, and most importantly, our study was conducted exclusively on a synthetic dataset. While we took significant steps to create a heterogeneous and challenging benchmark—incorporating diverse linguistic styles, a wide range of document lengths, and varying levels of clinical detail—this dataset cannot fully replicate the complexity of real-world clinical records. Specifically, our synthetic data, despite its variability, likely lacks the idiosyncratic shorthand, nonstandard abbreviations, ambiguities, and internal contradictions across multiple documents for a single patient that are common in authentic electronic health record data [26,27]. The high accuracy reported here may be, in part, an artifact of testing an LLM on relatively clean data generated by other LLMs. Consequently, our results should be interpreted as an upper-bound estimate of performance in a controlled environment. Even our error-injection simulation may not fully capture the nuanced challenges of real-world data. Therefore, rigorous validation on large-scale, multi-institutional, deidentified real-world datasets is the essential next step before the clinical deployment of this JIT paradigm can be considered.

Second, our study’s reliance on a single dataset of stage IV NSCLC raises questions about its generalizability. Although our validation on a smaller AML cohort showed consistent performance, the framework’s robustness across different cancer types, disease stages, and institutional documentation practices remains unproven and requires further validation. Third, our study used a single LLM architecture, and performance is prompt-dependent; comparative studies with other models (eg, CancerLLM [28]) and RAG frameworks are needed. Our preliminary tests showed that other leading frontier models (including from OpenAI and Anthropic) and local models could not handle this data volume, returning truncated or incomplete results. While our large-context method differs from alternative architectures like RAG, we recognize that the LLM landscape is rapidly evolving. Therefore, future comparative studies are needed to benchmark different approaches as their capabilities expand. Fourth, the autonomous generation of hypotheses, while compelling, must be treated as exploratory. Fifth, real-world deployment of our JIT paradigm faces certain ethical and practical hurdles. LLMs can perpetuate biases, creating ethical challenges that necessitate robust human oversight and model transparency to ensure accountability and health equity [29,30]. Therefore, responsible clinical integration will depend on developing clear regulatory frameworks and maintaining a human-in-the-loop approach to ensure patient safety. Human–AI interaction also requires real-world evaluation [31].

### Conclusion

In a controlled experiment using synthetic data, this study demonstrated that a frontier LLM can perform complex, multistep analytical tasks directly from unstructured text. The model successfully executed high-fidelity data extraction, answered multiconditional queries, and generated accurate end-to-end survival analyses. These results establish the technical feasibility of a JIT clinical analysis model under idealized conditions. The extent to which these capabilities generalize to the complexities of real-world clinical data remains unproven and requires rigorous, multi-institutional validation before any practical application can be considered.

## Supplementary material

10.2196/78332 Multimedia Appendix 1 Data scheme representing all variables for synthetic non–small cell lung cancer (n=240) and acute myeloid leukemia (n=80) cohorts.

10.2196/78332 Multimedia Appendix 2 Supplementary tables (S1-4) and figures (S1-5), prompts, and example letters.

## Abbreviations

AI: artificial intelligence
AML: acute myeloid leukemia
API: application programming interface
C-index: (Harrell’s) concordance index
GT: ground truth
HIPAA: Health Insurance Portability and Accountability Act
JIT: just-in-time
KRAS: Kirsten rat sarcoma viral oncogene homolog
LLM: large language model
MPE: multiparameter extraction
NSCLC: non–small cell lung cancer
OS: overall survival
PD-L1: programmed death-ligand 1
PFS: progression-free survival
RAG: retrieval-augmented generation
SPE: single parameter extraction
WT: wild type

## References

[1] Kefeli J, Berkowitz J, Acitores Cortina JM, Tsang KK, Tatonetti NP (2024). Generalizable and automated classification of TNM stage from pathology reports with external validation. Nat Commun.

[2] Swaminathan A, Ren AL, Wu JY (2024). Extraction of unstructured electronic health records to evaluate glioblastoma treatment patterns. JCO Clin Cancer Inform.

[3] Varma G, Yenukoti RK, Kumar M P (2025). A deep learning-enabled workflow to estimate real-world progression-free survival in patients with metastatic breast cancer: study using deidentified electronic health records. JMIR Cancer.

[4] Elmarakeby HA, Trukhanov PS, Arroyo VM (2023). Empirical evaluation of language modeling to ascertain cancer outcomes from clinical text reports. BMC Bioinformatics.

[5] Cohen AB, Adamson B, Larch JK, Amster G (2025). Large language model extraction of PD-L1 biomarker testing details from electronic health records. AI Precision Oncol.

[6] Chen D, Alnassar SA, Avison KE, Huang RS, Raman S (2025). Large language model applications for health information extraction in oncology: scoping review. JMIR Cancer.

[7] Wiest IC, Ferber D, Zhu J (2024). Privacy-preserving large language models for structured medical information retrieval. NPJ Digit Med.

[8] Yamagishi Y, Nakamura Y, Hanaoka S, Abe O (2025). Large language model approach for zero-shot information extraction and clustering of Japanese radiology reports: algorithm development and validation. JMIR Cancer.

[9] Hashtarkhani S, Rashid R, Brett CL (2025). Cancer diagnosis categorization in electronic health records using large language models and BioBERT: model performance evaluation study. JMIR Cancer.

[10] Yao J, Goldner E, Hochheiser H (2025). Systemic anticancer therapy timelines extraction from electronic medical records text: algorithm development and validation. JMIR Bioinform Biotech.

[11] Yao Y, Cen X, Gan L (2025). Automated esophageal cancer staging from free-text radiology reports: large language model evaluation study. JMIR Med Inform.

[12] Ferber D, Wiest IC, Wölflein G (2024). GPT-4 for information retrieval and comparison of medical oncology guidelines. NEJM AI.

[13] Xu R, Hong Y, Zhang F, Xu H (2024). Evaluation of the integration of retrieval-augmented generation in large language model for breast cancer nursing care responses. Sci Rep.

[14] Stuhlmiller TJ, Rabe A, Rapp J A scalable method for validated data extraction from electronic health records with large language models. Health Informatics.

[15] Hasan SMS, Rivera D, Wu XC, Durbin EB, Christian JB, Tourassi G (2020). Knowledge graph-enabled cancer data analytics. IEEE J Biomed Health Inform.

[16] Wang L, Li J, Zhuang B (2025). Accuracy of large language models when answering clinical research questions: systematic review and network meta-analysis. J Med Internet Res.

[17] Barr AA, Quan J, Guo E, Sezgin E (2025). Large language models generating synthetic clinical datasets: a feasibility and comparative analysis with real-world perioperative data. Front Artif Intell.

[18] Kehl KL, Groha S, Lepisto EM (2021). Clinical inflection point detection on the basis of EHR data to identify clinical trial-ready patients with cancer. JCO Clin Cancer Inform.

[19] Dahl S, Bøgsted M, Sagi T, Vesteghem C (2025). Performance of natural language processing for information extraction from electronic health records within cancer: systematic review. JMIR Med Inform.

[20] Van Veen D, Van Uden C, Blankemeier L (2024). Adapted large language models can outperform medical experts in clinical text summarization. Nat Med.

[21] Gauthier MP, Law JH, Le LW (2022). Automating access to real-world evidence. JTO Clin Res Rep.

[22] Elbattah M, Arnaud E, Ghazali DA, Dequen G, Dequen G (2024). Exploring the ethical challenges of large language models in emergency medicine: a comparative international review.

[23] Wiest IC, Verhees FG, Ferber D (2024). Detection of suicidality from medical text using privacy-preserving large language models. Br J Psychiatry.

[24] Lee D, Vaid A, Menon KM (2025). Using large language models to automate data extraction from surgical pathology reports: retrospective cohort study. JMIR Form Res.

[25] Wiest IC, Leßmann ME, Wolf F (2025). Deidentifying medical documents with local, privacy-preserving large language models: the LLM-anonymizer. NEJM AI.

[26] Savova GK, Danciu I, Alamudun F (2019). Use of natural language processing to extract clinical cancer phenotypes from electronic medical records. Cancer Res.

[27] Spasić I, Livsey J, Keane JA, Nenadić G (2014). Text mining of cancer-related information: review of current status and future directions. Int J Med Inform.

[28] Li M, Huang J, Yeung J, Blaes A, Johnson S, Liu H (2024). CancerLLM: a large language model in cancer domain. arXiv.

[29] Zhang K, Meng X, Yan X (2025). Revolutionizing health care: the transformative impact of large language models in medicine. J Med Internet Res.

[30] Verlingue L, Boyer C, Olgiati L, Brutti Mairesse C, Morel D, Blay JY (2024). Artificial intelligence in oncology: ensuring safe and effective integration of language models in clinical practice. Lancet Reg Health Eur.

[31] Wekenborg MK, Gilbert S, Kather JN (2025). Examining human-AI interaction in real-world healthcare beyond the laboratory. NPJ Digit Med.

[32] JIT-AI-registry. GitHub.

