# Supplementary material for: Enabling Just-in-Time Clinical Oncology Analysis With Large Language Models: Feasibility and Validation Study Using Unstructured Synthetic Data
Source: JMIR Med Inform. 2025 Dec 1;13:e78332. doi: 10.2196/78332 (PMC12670046; doi:10.2196/78332)
Supplement: Multimedia Appendix 2 [file medinform-v13-e78332-s002.docx]

**Supplementary Appendix A2**

Table of Contents

A2.1 Tables S1-4…………….........................………………......…………..….2

A2.2 Figures S1-5………….........................……...………………….……..….8

A2.3 Prompt for generation of letters……………………...………………….13

A2.4 Prompts for single parameter extraction………………...….………..….14

A2.5 Prompt for multiple parameter extraction…………………...……......…17

A2.6 Example prompt for question answering…………………….....……….18

A2.7 Example prompt for R code generation……………………...…….…....19

A2.8 Sample output from R code prompt………………………………...…...20

A2.9 Example prompt for finding contradictory or missing data……..………24

A2.10 Sample output for finding contradictory or missing data……………...25

A2.11 Prompt for AML multiple parameter extraction……………………….34

A2.12 Prompt for hypothesis generation……………………………………...35

A2.13 Example letter 1 (SYN007)………..…………………………………..37

A2.14 Example letter 2 (SYN020)……………..……………………………..46

A2.15 Example letter 3 (SYN092)…..………………………………………..48

A2.16 Example letter 4 (SYN117)..…………………………………………..49

**A2.1 Supplementary Tables**

**Table S1: Characteristics of 240 synthetic NSCLC reports and parameters included**. NSCLC=non-small cell lung cancer; PD-L1=programmed death ligand 1.

| **Length** | | **Median [range]** |
| --- | --- | --- |
|  | Words | 582 [109-3,147] |
|  | Pages | 2 [1-16] |
| **Type of letter** | | **N** |
|  | Case summary | 23 |
|  | Clinic note | 20 |
|  | Consultation note | 19 |
|  | Death note | 34 |
|  | Discharge summary | 91 |
|  | Infusion note | 7 |
|  | Progress note | 46 |
| **Parameters** | | **Description** |
| 1 | ID | SYN001-SYN240 |
| 2 | Name | surname, first name(s) |
| 3 | Date of birth | YYYY-MM-DD |
| 4 | Gender | m/f |
| 5 | Date of diagnosis | YYYY-MM |
| 6 | Distant metastases at diagnosis | 7 sites with 1-3 per patient |
| 7 | PD-L1 status | <1%, 1-49%, ≥50% |
| 8 | Driver mutation | 11 mutations |
| 9 | Date of initial treatment | YYYY-MM |
| 10 | First-line treatment | 13 treatment types |
| 11 | Progressed on first-line | yes/no |
| 12 | Alive | yes/no |
| 13 | Ongoing on first-line | yes/no |
| 14 | Progression-free survival (PFS) | months (+/-1) |
| 15 | Overall survival (OS) | months (+/-1) |

**Table S2: Characteristics of 80 synthetic AML reports and parameters included**. AML=acute myeloid leukemia; ELN=European LeukemiaNet.

| **Length** | | **Median [range]** |
| --- | --- | --- |
|  | Words | 455 [104-1,114] |
|  | Pages | 2 [1-6] |
| **Type of letter** | | **N** |
|  | Case summary | 13 |
|  | Clinic/progress note | 21 |
|  | Consultation note | 5 |
|  | Death note | 12 |
|  | Discharge summary | 21 |
|  | Email | 6 |
|  | Mix of documents | 2 |
| **Parameters** | | **Description** |
| 1 | ID | AML001-AML080 |
| 2 | Name | surname, first name(s) |
| 3 | Date of birth | YYYY-MM-DD |
| 4 | Gender | m/f |
| 5 | Date of diagnosis | YYYY-MM |
| 6 | ELN risk classification | favorable, intermediate, adverse |
| 7 | Mutations | 36 mutations incl. combinations |
| 8 | Allogeneic transplantation | planned, done, no |
| 9 | Date of initial treatment | YYYY-MM |
| 10 | First-line treatment | 13 treatment types |
| 11 | Progressed on first-line | yes/no |
| 12 | Alive | yes/no |
| 13 | Ongoing on first-line | yes/no |
| 14 | Progression-free survival (PFS) | months (+/-1) |
| 15 | Overall survival (OS) | months (+/-1) |

**Table S3: Description of injected errors**. AUC=area under the curve; CT=computed tomography; IC=immune cell (score); NSCLC=non-small cell lung cancer; PD-L1=programmed death-ligand 1; SCLC=small cell lung cancer; TPS=tumor proportion score; WT=wild type.

| **ID** | **Typographical error** | **Ambiguity/Contradiction** | **Missing data field** |
| --- | --- | --- | --- |
| SYN001 | EGFR -> EFGR | Date of diagnosis: 22.07.2021 -> 2022 | PD-L1 status |
| SYN002 | hepatic -> heptic | PD-L1 low vs. 75% | Date of treatment initiation |
| SYN003 | Pembrolizumab -> Pemborlizumab | Name: Wei -> Schmidt | Mutations |
| SYN004 | female -> fmale | Treatment start 2023 -> 2022 | Date of birth |
| SYN005 | Dec 9, 2021 -> Dec 9, 20221 | Mutation status: pending vs. negative | Treatment |
| SYN006 | Osimertinib -> Ostimertinib | Mrs. XY, male patient | Metastases |
| SYN007 | stable disease -> stalbe disease | No driver mutations vs. ALK rearrangement | Name |
| SYN008 | September -> Setember | Name of doctor | PD-L1 status |
| SYN009 | TPS -> PTS | Date of birth 1957 -> 1997 | Treatment |
| SYN010 | BRAF -> BARF | Hepatic metastases (femur, rip, ...) | Date of treatment initiation |
| SYN011 | June -> Jne | NSCLC -> SCLC | Mutations |
| SYN012 | Carboplatin -> Caboplatin | Cycle 20 -> 2 | Date of birth |
| SYN013 | PD-L1 -> PD-1 | Date of death 2025 -> 2024 | Treatment |
| SYN014 | lymphadenopathy -> lymphdenopathy | EGFR allele frequency 0.35% | Date of diagnosis |
| SYN015 | 69-year -> 9-year | First-line docetaxel | PD-L1 status |
| SYN016 | partial response -> partial reponse | EGFR/ALK/ROS1/BRAF neg. -> pos. | Name |
| SYN017 | brain metastases -> brain mestatases | Date of birth 9/7/48 -> 9/7/4 | Treatment |
| SYN018 | wild type -> wlid type | Treatment start: August -> April | ID |
| SYN019 | January -> Jarnury | TPS <1% -> 0100% | Date of birth |
| SYN020 | Adrenal -> Adernal | Date of diagnosis 2019 -> 2009 | Mutations |
| SYN021 | EGFR mutation positive -> postive | Added: CT scan (June 12, 2022): unequivocal, potentially progression. | Treatment |
| SYN022 | death -> daeth | Treatment from Mar-Dec 2021 -> Dec->Mar 2021 | PD-L1 status |
| SYN023 | PD-L1 negative -> negtive | Carbo/Pembro/Pembrolizumab | Date of diagnosis |
| SYN024 | NTRK -> NRTK | Larotrectinib -> Entrectinib on first mention | Date of letter |
| SYN025 | 2021 -> 2012 | First-line Pembrolizumab instead of Carbo/Pem/Pembro | Date of birth |
| SYN026 | alectinib -> alctinib | Stage IV -> II (no metastases) | Date of diagnosis |
| SYN027 | WT -> TW | First-line treatment until Aug 2021 -> 2022 | Name |
| SYN028 | exon -> exno | Date of diagnosis 2023 -> 2024 | PD-L1 status |
| SYN029 | Gender: M -> Gender: N | KRAS G12C -> G12D | ID |
| SYN030 | monotherapy -> monthreapy | Several first-line treatments: Pembrolizumab, Alectinib | Date of birth |
| SYN031 | osseous -> osoeus | Date of initiation 1L 2022 -> 2023 | Mutations |
| SYN032 | initial diagnosis -> intial diangosis | PD-L1 >50 (actual score 5%) | Mutations |
| SYN033 | pleural -> pleual | Gender M -> F | Date of letter |
| SYN034 | ALK -> AKL | On Alectinib x ~2,5 years -> 1,5 years | Date of birth |
| SYN035 | Pemetrexed -> Pemtrexed | WT (ROS1 rearrangement) | Name |
| SYN036 | ROS1 -> ORS1 | Entrectinib with Larotrectinib dose | PD-L1 status |
| SYN037 | October 2020 -> October 220 | Carboplatlin -> Cisplatin (AUC 5) | Metastases |
| SYN038 | pulmonary -> pulomonary | PD-L1 status: TPS and IC mixed up | Date of letter |
| SYN039 | May -> My | Added: "New hepatic lesions, highly suspicious for metastases." | Date of birth |
| SYN040 | PD-L1-high -> PD-L1-hgih | Several patient names | Mutations |

**Table S4: Detailed evaluation of hypothesis generation process.** CIT=chemo-immunotherapy; CNS=central nervous system; irAE=immune-related adverse events, LDH=lactate dehydrogenase; NSCLC=non-small cell lung-cancer; PD-L1=programmed death-ligand 1; PFS=progression-free survival; TKI=tyrosine kinase inhibitor; TPS=tumor proportion score; WT=wild type.

| **Hypothesis** | **Generated explanation** | **Comment** |
| --- | --- | --- |
| N=80 | | |
| H1: In patients with KRAS-mutated NSCLC treated with an immune checkpoint inhibitor-containing regimen, the presence of a concurrent STK11 loss-of-function mutation is associated with a significantly increased risk of developing severe (Grade 3) immune-mediated colitis. | 12 KRAS-mutated patients: 3 with STK11-comutation, 9 without. In KRAS/STK11 co-mutated group 2 out of 3 developed immune-mediated colitis, vs. 0 out of 9 in the KRAS-mutated, STK11-WT group. developed colitis | Counter-intuitive: STK11-deficient tumors tend to have reduced T-cell inflammation. |
| H2: In patients with driver-negative, PD-L1-high (TPS ≥50%) NSCL, the development of Grade ≥2 immune-mediated hepatitis during first-line pembrolizumab monotherapy is associated with a uniquely durable and profound clinical response compared to patients who experience other immune-related adverse events (irAEs) or no irAEs. | 11 patients with WT, PD-L1 >50% with first-line pembrolizumab: 3 with grade 2+ hepatitis, 8 without. The 3 patients with hepatitis all have ongoing response >30 months vs. 5 out 8 progression after 14-20 months and 3 with ongoing response <18 months | While many studies link immune-related adverse evenets and outcome, there is no consistent evidence for hepatitis as a superior predictor. |
| H3: In patients with Stage IV EGFR-mutant NSCLC treated with first-line Osimertinib, those with an L858R point mutation have a higher propensity to develop carcinomatous meningitis as a site of disease progression compared to patients with an Exon 19 deletion. | 13 patients with EGFR-mutated patients treated with osimertinib: 6 with L858R mutation and 7 with Exon 19 deletion. 2 out 6 patients with L858R mutation developed CNS relapse vs. 0 out of 7 in Exon 19 deletion group. | Multiple studies have reported higher CNS risk with L858 vs Exon 19 deletion, but not specifically for progression pattern under osimertinib. |
| H4: In patients with NSCLC harboring a MET Exon 14 skipping mutation, the development of Grade ≥2 peripheral edema during first-line therapy with a MET tyrosine kinase inhibitor is strongly associated with concurrent, treatment-emergent hypoalbuminemia. | 4 patients with MET Exon 14 skipping mutation and first-line TKI: 3 with edema, 1 without. 2 out 3 edema patient had concurrent hypoalbuminemia vs. 0 out 1 without edema. | Edema is well-known class effect of capmatinib/tepotinib, and some works have suggested association with hypalbuminemia. |
| H5: In patients with RET fusion-positive NSCLC, treatment with selective RET tyrosine kinase inhibitors is associated with a high incidence of a spectrum of cardiovascular toxicities, including but not limited to hypertension, suggesting a class effect that warrants proactive cardiac monitoring beyond routine blood pressure checks. | 3 patients with RET fusion positive NSCLC on RET-targeting TKI. 3 out 3 developed hypertension, one had atrial fibrillation and one had QTc prolongation. | Hypertension is widely recognized as RET TKI side effect, and some evidence exists on QT prolongation. |
| N=160 | | |
| H6: In patients with EGFR-mutant NSCLC treated with first-line osimertinib, the presence of an L858R point mutation is associated with an increased risk of developing clinically significant (Grade ≥2) drug-induced pneumonitis compared to patients with an Exon 19 deletion. | 25 patients with EGFR mutation: 13 with L858R and 12 with Exon 19 deletion. 2 out 13 L858R-mutated patients developed pneumonitis vs. 0 out of 12 in Exon 19 deletion. | No obvious mechanistic pathway for causal link between tumor genotype and lung toxicity. |
| H7: In patients with KRAS-mutant NSCLC treated with first-line platinum-doublet chemotherapy plus pembrolizumab, the presence of a co-occurring STK11 mutation is associated with a higher rate of primary progressive disease and shorter duration of benefit compared to patients with STK11 wild-type tumors. | 9 KRAS-mutated patients with first-line CIT: 3 with STK11 co-mutation, 6 without. 3 out of 3 patients progressed <12 months in co-mutations group vs. 2 out 6 in STK11 WT group. | Multiple retrospective analyses report inferior outcomes with STK11 |
| H8: In patients with Stage IV ALK-rearranged Non-Small Cell Lung Adenocarcinoma treated with first-line alectinib, the presence of brain metastases at diagnosis (managed with upfront stereotactic radiosurgery) is associated with an increased risk of developing subsequent intracranial-only progression compared to patients who present without brain metastases. | 16 ALK-rearranged patients: 7 with brain metastases at diagnosis, 9 without. 1 out of 7 developed CNS progression in brain metastasis group vs. 0 out of 9. | Number of events (1 vs. 0) is very small. Effect has been shown in many trials. |
| H9: In patients with driver-negative (pan-wild-type) NSCLC and low PD-L1 expression (TPS <1%), the presence of liver metastases at diagnosis is associated with a significantly shorter progression-free survival (PFS < 6 months) on first-line platinum-doublet chemotherapy plus pembrolizumab compared to patients without liver metastases. | 8 WT patients with PD-L1 <1%: 3 with liver metastases at diagnosis, 5 without. 2 out of 3 patients with liver metastases progressed with a PFS <6 months vs. 0 out of 5 patients with PFS <6 months in no liver metastases group. | It is well known that liver metastases predict worse outcomes with ICI. |
| H10: In patients with driver-negative (pan-wild-type) NSCLC and low-to-intermediate PD-L1 expression (TPS < 50%), an elevated baseline Lactate Dehydrogenase (LDH) level is associated with a higher rate of primary progressive disease on first-line platinum-doublet chemotherapy plus pembrolizumab. | 11 WT patients with PD-L1 <50% treated with CIT: 3 with elevated LDH and 8 with normal LDH. 2 out of 3 patients with elevated LDH experienced primary progressive disease vs. 0 out of 8. | Emerging data suggests LDH as prognostic marker for CIT. |

**A2.2 Supplementary Figures**

**
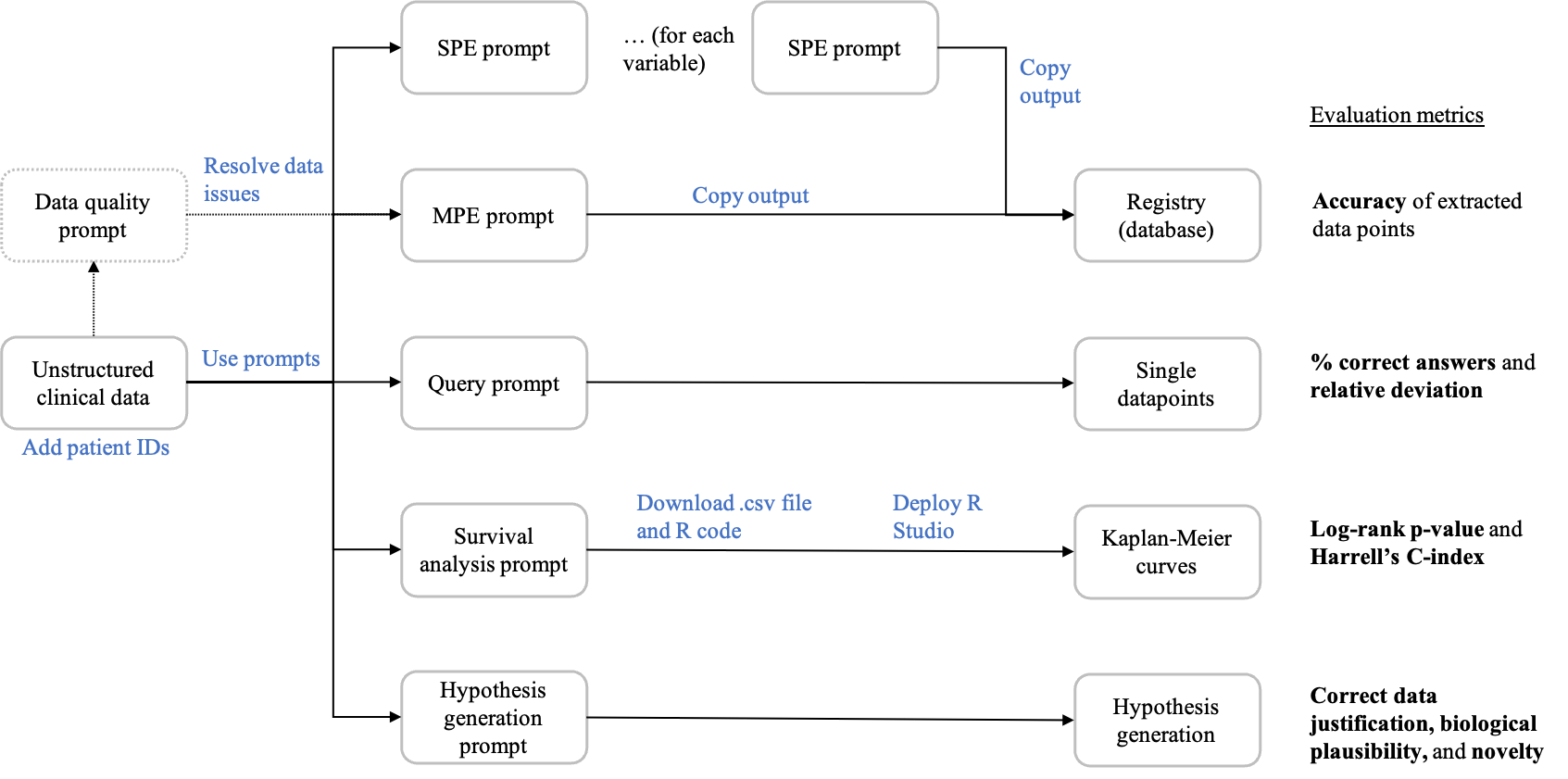
**

**Figure S1: Flowchart depicting experimental design.** Dark blue text represents actions taken by humans.

**Figure S2**

**Figure S2: Confusion matrices under multiparameter extraction.** Example confusion matrices for 'Alive status' (left) and 'Progression status' (right) under multiparameter extraction for the 1×240 batch. The diagonal cells (top-left to bottom-right) represent correct predictions, while off-diagonal cells represent misclassifications. Each cell provides the absolute number of cases and the row-wise percentage (dark blue 100%, white 0%).

**Figure S3: Evaluation Processing Time and Information Extraction accuracy.** **(A)** and **(B)** Stacked bars representing time taken in seconds by LLM to process one individual parameter or all n=14 parameters together with different batching strategies. **(C)** Heatmap showing the accuracy across different clinical variables (columns) and data batching strategies (rows) of LLM on single parameter extraction. Green indicates high accuracy (100%), yellow medium (95%), and red low accuracy (90%), Y-axis depicts whether all 240 were processed simultaneously or in sets of 2, 3, 4 or 6. LLM=large language model; SPE=single parameter extraction; MPE=multiparameter extraction; DOB=date of birth; PFS=progression-free survival; OS=overall survival.

**Figure S4: Harrell's Concordance Index for LLM-Extracted vs. Ground-Truth Survival Data.** The plot displays the C-index for Overall Survival (OS, left) and Progression-Free Survival (PFS, right) from n=12 independent analysis repetitions. A C-index of 1.0 signifies perfect agreement and 0.0 perfect disagreement between two survival models. The dotted line at 0.5 indicates no statistical difference between the model based on LLM-extracted data and the model based on ground-truth data.

**Figure S5: LLM performance on small acute myeloid leukemia dataset (N=80).** **(A)** Clinical questions requiring aggregation of 1-3 parameters. **(B)** The percentage of correct replicates and **(C)** the relative deviation in direct question-answering from unstructured text, using 12 replicates per question **(D)** Automated Kaplan-Meier curve for Overall Survival (OS) and **(E)** Progression-Free Survival (PFS), comparing LLM-extracted data against the ground truth, including number at risk tables and log-rank tests. Shaded areas represent 95% confidence intervals. LLM=large language model.

**A2.3 Prompt for generation of letters**

You are a specialist in creating discharge notes.

For each of the following patients create a discharge note. Make sure that every note is of individual length, style and depth of detail. Use the given parameters as starting points, but use creativity. for example add comorbidites and medication variably. put the patient in further lines of treatment where applicable (only 1L is defined). do not specifically define the pfs, but it should be intrinsic in the data. each letter should be between 300-2000 words, can include lots of lab data, imaging or pathology reports etc. give specific value for pdl1 (and include tps cps ic scores).

Patient_ID,DOB,Gender,Date_of_Diagnosis,Site_of_Metastases,Driver_Mutation,PDL1_Status_TPS,First_Line_Treatment,Start_of_1L_Treatment,PFS_months_or_Ongoing,OS_months_or_Ongoing

SYN026,1964-01-24,Female,2021-04-12,"Bone, Brain",ALK fusion,<1%,Alectinib,2021-05-03,Ongoing,Ongoing

SYN027,1947-11-11,Male,2019-08-22,"Lung, Liver",WT,>=50%,Pembrolizumab,2019-09-13,18 months,40 months

SYN028,1972-05-06,Female,2023-06-07,"Adrenal",EGFR Exon 19 del,1-49%,Osimertinib,2023-06-29,Ongoing,Ongoing

SYN029,1953-08-29,Male,2022-02-17,"Bone, Lymph Nodes",KRAS G12D,<1%,Carbo/Pem/Pembro,2022-03-11,13 months,Ongoing

SYN030,1960-12-15,Female,2021-09-28,"Liver",WT,>=50%,Pembrolizumab,2021-10-20,Ongoing,Ongoing

**A2.4 Prompts for single parameter extraction**

You are an expert at extracting medical information from text.

Create a table with the following parameters:

- ID (or MRN)

- Name (Surname, First name(s))

You are an expert at extracting medical information from text.

Create a table with the following parameters:

- ID (or MRN)

- Date of birth (YYYY-MM-DD)

You are an expert at extracting medical information from text.

Create a table with the following parameters:

- ID (or MRN)

- Gender (m/f)

You are an expert at extracting medical information from text.

Create a table with the following parameters:

- ID (or MRN)

- Date of diagnosis (YYYY-MM)

Use exact dates and make sure to extract the correct date (cross-check with other dates in the letter to make sure you use the correct dd/mm!).

You are an expert at extracting medical information from text.

Create a table with the following parameters:

- ID (or MRN)

- Site of distant metastasis at first diagnosis [Brain, Adrenal, Pleura, Bone, Liver, Lung (ipsi- or contralateral), Lymph nodes (only if non-local, i.e. not hilar, mediastinal, subclavicular etc.)]

You are an expert at extracting medical information from text.

Create a table with the following parameters:

- ID (or MRN)

- Driver mutation (ALK fusion, BRAF V600E, EGFR Exon 19 del, EGFR L858R, KRAS G12C, KRAS G12D, KRAS G12V, MET Exon 14 skip, NTRK fusion, ROS1 fusion, RET fusion, WT, n/a)

You are an expert at extracting medical information from text.

Create a table with the following parameters:

- ID (or MRN)

- Date of initiation of 1L treatment (YYYY-MM)

Use exact dates and make sure to extract the correct date (cross-check with other dates in the letter to make sure you use the correct dd/mm!).

You are an expert at extracting medical information from text.

Create a table with the following parameters:

- ID (or MRN)

- 1L treatment (Alectinib, Capmatinib, Carbo/Paclitaxel, Carbo/Paclitaxel/Pembrolizumab, Carbo/Pem/Pembro, Dabrafenib+Trametinib, Entrectinib, Larotrectinib, Osimertinib, Pembrolizumab, Pralsetinib, Selpercatinib, Tepotinib)

You are an expert at extracting medical information from text.

Create a table with the following parameters:

- ID (or MRN)

- 1L treatment ongoing? (y=ongoing, n=stopped permanently)

You are an expert at extracting medical information from text.

Create a table with the following parameters:

- ID (or MRN)

- progression on 1L treatment (0=no progression, 1=progression)

You are an expert at extracting medical information from text.

Create a table with the following parameters:

- ID (or MRN)

- Alive (0=alive, 1=dead)

You are an expert at extracting medical information from text.

Create a table with the following parameters:

- ID (or MRN)

- PFS since start of 1L treatment in months

Use exact dates and make sure to extract the correct date (cross-check with other dates in the letter to make sure you use the correct dd/mm!).

if no exact date provided assume 1st of months (and if early 1st, if mid 15th and if end 30th).

Definition of PFS: time from treatment start to disease progression (irrespective of treatment continuation) or last known time point alive without progression

Definition of OS: time from treatment start to death or last known time point alive

example:

letter for SYN248 from 2024-03-25, initiation February 2023, still alive and on treatment without progression

| Patient MRN | 1L treatment ongoing (y/n) | progression on 1L (0=no, 1=yes) | PFS since start of 1L (months) | alive (0=alive, 1=dead) | OS since start of 1L (months) |

| SYN248 | y | 0 | 14 | 0 | 14 |

For PFS and OS only use full integer months, i.e. 15 months + 13 days = 15 months and 15 months + 17 days = 16 months

You are an expert at extracting medical information from text.

Create a table with the following parameters:

- ID (or MRN)

- OS since start of 1L treatment in months

Use exact dates and make sure to extract the correct date (cross-check with other dates in the letter to make sure you use the correct dd/mm!).

if no exact date provided assume 1st of months (and if early 1st, if mid 15th and if end 30th).

Definition of PFS: time from treatment start to disease progression (irrespective of treatment continuation) or last known time point alive without progression

Definition of OS: time from treatment start to death or last known time point alive

example:

letter for SYN248 from 2024-03-25, initiation February 2023, still alive and on treatment without progression

| Patient MRN | 1L treatment ongoing (y/n) | progression on 1L (0=no, 1=yes) | PFS since start of 1L (months) | alive (0=alive, 1=dead) | OS since start of 1L (months) |

| SYN248 | y | 0 | 14 | 0 | 14 |

For PFS and OS only use full integer months, i.e. 15 months + 13 days = 15 months and 15 months + 17 days = 16 months

**A2.5 Prompt for multiple parameter extraction**

You are an expert at extracting medical information from text.

Create a table with the following parameters:

- ID (or MRN)

- Name (Surname, First name(s))

- Date of birth (YYYY-MM-DD)

- Gender (m/f)

- Date of diagnosis (YYYY-MM)

- Site of distant metastasis at first diagnosis [Brain, Adrenal, Pleura, Bone, Liver, Lung (ipsi- or contralateral), Lymph nodes (only if non-local, i.e. not hilar, mediastinal, subclavicular etc.)]

- PD-L1 status (<1%, 1-49%, >=50%)

- Driver mutation (ALK fusion, BRAF V600E, EGFR Exon 19 del, EGFR L858R, KRAS G12C, KRAS G12D, KRAS G12V, MET Exon 14 skip, NTRK fusion, ROS1 fusion, RET fusion, WT, n/a)

- Date of initiation of 1L treatment (YYYY-MM)

- 1L treatment (Alectinib, Capmatinib, Carbo/Paclitaxel, Carbo/Paclitaxel/Pembrolizumab, Carbo/Pem/Pembro, Dabrafenib+Trametinib, Entrectinib, Larotrectinib, Osimertinib, Pembrolizumab, Pralsetinib, Selpercatinib, Tepotinib)

- 1L treatment ongoing? (y=ongoing, n=stopped permanently)

- progression on 1L treatment (0=no progression, 1=progression)

- Alive (0=alive, 1=dead)

- PFS since start of 1L treatment in months

- OS since start of 1L treatment in months

Use exact dates and make sure to extract the correct date (cross-check with other dates in the letter to make sure you use the correct dd/mm!).

if no exact date provided assume 1st of months (and if early 1st, if mid 15th and if end 30th).

Definition of PFS: time from treatment start to disease progression (irrespective of treatment continuation) or last known time point alive without progression

Definition of OS: time from treatment start to death or last known time point alive

example:

letter for SYN248 from 2024-03-25, initiation February 2023, still alive and on treatment without progression

| Patient MRN | 1L treatment ongoing (y/n) | progression on 1L (0=no, 1=yes) | PFS since start of 1L (months) | alive (0=alive, 1=dead) | OS since start of 1L (months) |

| SYN248 | y | 0 | 14 | 0 | 14 |

For PFS and OS only use full integer months, i.e. 15 months + 13 days = 15 months and 15 months + 17 days = 16 months

**A2.6 Example prompt for question answering**

You are an expert at extracting medical information from text.

Task: how many patients are born before 1970, are/were on Pembrolizumab containing 1L therapy AND have a KRAS mutation? (all 3 conditions fulfilled)

Rules:

Use exact dates and make sure to extract the correct date (cross-check with other dates in the letter to make sure you use the correct dd/mm!).

if no exact date provided assume 1st of months (and if early 1st, if mid 15th and if end 30th).

Definition of PFS: time from treatment start to disease progression (irrespective of treatment continuation) or last known time point alive without progression

Definition of OS: time from treatment start to death or last known time point alive

**A2.7 Example prompt for R code generation**

You are an expert at extracting medical information from text.

Task: Generate a Kaplan-Meier survival curve for OS for all 160 patients from your extracted data by

1) creating a table with the columns ID, Time, Event and one patient per row. The user will then download the table as a csv file ("OS160extracted.csv") into the folder within R.

2) creating an R code that compares "OS160extracted.csv" to the ground truth (which is already saved done and saved under "OS160original.csv") using log-rank test, showing both curves in the diagram with different colours.

Rules:

* Definition of OS: time from treatment start to death or last known time point alive

* Use exact dates and make sure to extract the correct date (cross-check with other dates in the letter to make sure you use the correct dd/mm!).

if no exact date provided assume 1st of months (and if early 1st, if mid 15th and if end 30th).

* For OS only use full integer months, i.e. 15 months + 13 days = 15 months and 15 months + 17 days = 16 months

**A2.8 Example output for R code prompt**

Here is the survival data extracted from the patient records.

**1) Extracted Survival Data Table**

code Csv

downloadcontent_copy

expand_less

ID,Time,Event

SYN001,19,0

SYN002,16,0

SYN003,35,1

SYN004,6,0

SYN005,12,0

SYN006,32,0

SYN007,33,0

SYN008,18,0

SYN009,9,0

SYN010,14,0

SYN011,21,0

SYN012,16,0

SYN013,35,1

SYN014,3,0

SYN015,13,1

SYN016,10,0

SYN017,54,0

SYN018,8,0

SYN019,51,0

SYN020,13,1

SYN021,25,1

SYN022,19,1

SYN023,20,1

SYN024,8,0

SYN025,11,0

SYN026,48,0

SYN027,29,1

SYN028,22,0

SYN029,37,0

SYN030,42,0

SYN031,15,0

SYN032,68,1

SYN033,1,0

SYN034,30,0

SYN035,11,0

SYN036,23,0

SYN037,20,1

SYN038,28,0

SYN039,4,0

SYN040,5,0

SYN041,12,0

SYN042,6,0

SYN043,35,1

SYN044,6,0

SYN045,36,0

SYN046,42,0

SYN047,34,0

SYN048,7,0

SYN049,15,1

SYN050,32,0

SYN051,12,1

SYN052,50,0

SYN053,2,0

SYN054,5,0

SYN055,19,0

SYN056,18,0

SYN057,42,0

SYN058,7,0

SYN059,16,1

SYN060,8,0

SYN061,28,1

SYN062,3,0

SYN063,37,0

SYN064,9,0

SYN065,28,0

SYN066,2,0

SYN067,14,1

SYN068,12,0

SYN069,19,1

SYN070,27,0

SYN071,20,0

SYN072,5,0

SYN073,25,1

SYN074,8,0

SYN075,37,0

SYN076,7,0

SYN077,42,1

SYN078,11,1

SYN079,42,0

SYN080,23,0

SYN081,22,1

SYN082,15,0

SYN083,29,0

SYN084,29,0

SYN085,21,0

SYN086,51,1

SYN087,43,0

SYN088,9,0

SYN089,54,1

SYN090,0,0

SYN091,16,0

SYN092,5,0

SYN093,26,0

SYN094,14,0

SYN095,20,0

SYN096,20,0

SYN097,33,1

SYN098,11,0

SYN099,24,0

SYN100,8,0

SYN101,23,0

SYN102,20,1

SYN103,34,1

SYN104,5,0

SYN105,15,0

SYN106,13,0

SYN107,13,1

SYN108,19,0

SYN109,50,1

SYN110,19,0

SYN111,34,0

SYN112,7,0

SYN113,33,1

SYN114,22,0

SYN115,13,0

SYN116,6,0

SYN117,49,1

SYN118,29,0

SYN119,18,1

SYN120,21,0

SYN121,23,0

SYN122,17,0

SYN123,24,1

SYN124,2,0

SYN125,2,1

SYN126,16,0

SYN127,45,0

SYN128,24,0

SYN129,57,1

SYN130,30,0

SYN131,18,1

SYN132,13,0

SYN133,43,0

SYN134,1,0

SYN135,38,0

SYN136,28,0

SYN137,46,0

SYN138,20,0

SYN139,30,1

SYN140,37,0

SYN141,13,1

SYN142,21,1

SYN143,10,1

SYN144,12,0

SYN145,2,0

SYN146,20,0

SYN147,41,1

SYN148,8,0

SYN149,24,0

SYN150,25,0

SYN151,26,0

SYN152,11,1

SYN153,31,1

SYN154,7,0

SYN155,1,0

SYN156,7,0

SYN157,16,1

SYN158,6,0

SYN159,20,1

SYN160,23,0

**2) R Code for Kaplan-Meier Curve Generation and Comparison**

To use this code, save the table above as a CSV file named OS160extracted.csv in your R working directory. Ensure the ground truth file OS160original.csv is also present in the same directory.

code R

downloadcontent_copy

expand_less

# Install and load necessary libraries

# install.packages(c("survival", "survminer"))

library(survival)

library(survminer)

# --- Load Data ---

# Load the extracted data

extracted_data <- read.csv("OS160extracted.csv")

# Load the ground truth data

original_data <- read.csv("OS160original.csv")

# --- Combine Data for Comparison ---

# Add a 'source' column to each dataframe to identify the origin

extracted_data$source <- 'Extracted'

original_data$source <- 'Original'

# Combine the two dataframes into a single one

combined_data <- rbind(extracted_data, original_data)

# --- Create a Single Survival Object Stratified by Source ---

# This is the standard and preferred way to compare two groups in survminer

fit_combined <- survfit(Surv(Time, Event) ~ source, data = combined_data)

# --- Plot the Kaplan-Meier Curves with Automatic P-value ---

# By using a single stratified fit object, ggsurvplot can now automatically

# calculate and display the log-rank p-value by setting pval = TRUE.

ggsurvplot(

fit_combined,

data = combined_data,

pval = TRUE, # This will now work correctly

conf.int = TRUE,

palette = c("#E7B800", "#2E9FDF"), # Custom colors for "Extracted" and "Original"

risk.table = TRUE,

risk.table.col = "strata", # Color risk table rows by group

risk.table.y.text.col = TRUE,

risk.table.y.text = FALSE,

tables.height = 0.2,

legend.labs = c("Extracted Data", "Ground Truth"),

legend.title = "Dataset",

title = "Kaplan-Meier Curve for Overall Survival (OS)",

subtitle = "Comparison of Extracted Data vs. Ground Truth",

xlab = "Time in Months",

ylab = "Overall Survival Probability",

ggtheme = theme_minimal(),

surv.median.line = "hv" # Add median survival lines

)

**A2.9 Prompt for finding contradictory or missing data**

*Normal prompt for multiparameter extraction (see A2.5)*

+

Before generating the final table, perform a Critical Data Audit. Your goal is to act as a clinical data manager and identify every piece of information that is missing, ambiguous, or logically inconsistent. This audit is the most important part of your task.

Present this audit in a structured list under three distinct headings:

1. Missing Data Points:

- List every field from the table for which no information can be found in the source text or for which data is incomplete - leading to n/a, XXX. For calculated fields like PFS and OS, you must be extremely precise.
- Directly Missing: Explicitly state which primary data point is missing. (e.g., "Date of birth is not mentioned in the text.")
- Assumptions necessary: Explicitly state if data is incomplete and you have to assume something!

2. Ambiguous, Vague, or Contradictory Data:

- List any data point that is mentioned but is not precise enough to fit the required format or categories. For each item, state the ambiguity and pose a clarifying question.
- Vague Dates: The text mentions a time period but not a specific date that can be resolved by the "1st/15th/30th" rule.
  - Example: "1L treatment initiation date: Text says 'started therapy last winter'. This is ambiguous. Does 'last winter' refer to Dec 2023, Jan 2024, or Feb 2024?"
- Unclear Categorization: The text uses terminology that doesn't map directly to the allowed categories.
  - Example: "PD-L1 status: Text states 'PD-L1 positive'. This is ambiguous as it does not fit into the required categories of '<1%', '1-49%', or '>=50%'."
  - Example: "Site of distant metastasis: Text mentions 'spread to lymph nodes' but does not specify if they are non-local (e.g., supraclavicular) or local (e.g., hilar, mediastinal), which is required by the definition."
- Conflicting Information: The text provides multiple pieces of information that could refer to the same data point.
  - Example: "Date of diagnosis: The initial consultation note is dated 2022-08-15, but a pathology report confirming the diagnosis is dated 2022-08-22. Which one should be used as the Date of Diagnosis?"
- Timeline Errors: Check if dates are in a logical sequence.
  - Example: "Progression on 1L treatment: The date of progression (2023-05-10) is listed as being before the date of 1L treatment initiation (2023-07-20). This is a logical contradiction."
- Status Inconsistencies: Check if different status fields conflict with each other.
  - Example: "Alive / 1L treatment ongoing: The patient is listed as 'Dead' (Alive=1), but the '1L treatment ongoing?' field is marked 'y'. A deceased patient cannot have ongoing treatment."

For every n/a, XXX, or empty field in your final table, there must be a corresponding entry in this Critical Data Audit. After presenting this complete audit, you may proceed to create the data table.

**A2.10 Sample output for finding contradictory of missing data**

Here is the requested medical information extraction.

**Critical Data Audit**

This audit identifies missing, ambiguous, or logically inconsistent data points from the source documents for each patient record.

**SYN001 (Miller, Robert James)**

1. **Missing Data Points:**
   - **PD-L1 status:** Not mentioned in the text.
2. **Ambiguous, Vague, or Contradictory Data:**
   - **Date of diagnosis:** The text states diagnosis was on "July 22, 2022". However, the entire clinical timeline (Initial Staging July 2021, Pathology 07/25/2021, 1L Treatment Start 08/10/2021) indicates the diagnosis occurred in 2021. The 2022 date is a logical contradiction. **Action Taken:** The pathology report date of 2021-07-25 was used as the date of diagnosis.

**SYN002 (Vasquez, Elena)**

1. **Missing Data Points:**
   - **Date of initiation of 1L treatment:** The exact start date for Pembrolizumab is not provided.
   - **Assumptions necessary for PFS/OS:** The start date was estimated as 2022-12-15 based on the treatment duration of "22 cycles" (q3wks) prior to the report date of 2024-04-13. This assumption directly impacts the PFS and OS calculations.
2. **Ambiguous, Vague, or Contradictory Data:**
   - **PD-L1 status:** The text is contradictory, stating "PD-L1 Expression: low" in one section and "Tumor Proportion Score 75%" in another. A TPS of 75% is considered high expression, not low. **Action Taken:** The specific quantitative value (75%) was used, categorizing it as '>=50%'.

**SYN003 (Chen, David Wei)**

1. **Missing Data Points:**
   - **Driver mutation:** Molecular testing results for driver mutations are not mentioned. **Action Taken:** Assumed to be Wild-Type (WT).
2. **Ambiguous, Vague, or Contradictory Data:**
   - **Name:** The patient identification block lists the name as "Chen, David Wei," but the narrative text refers to the patient as "Mr. Schmidt." **Action Taken:** The name from the patient identification block (Chen) was used.
   - **Date of diagnosis:** The text states "May 2020." **Action Taken:** Per the rule, the 1st of the month (2020-05-01) was used.
   - **Date of initiation of 1L treatment:** The text states "June 2020." **Action Taken:** Per the rule, the 1st of the month (2020-06-01) was used.
   - **Date of progression on 1L treatment:** The text states "August 2021." **Action Taken:** Per the rule, the 1st of the month (2021-08-01) was used.

**SYN004 (Jenkins, Sarah Lynn)**

1. **Missing Data Points:**
   - **Date of birth:** Not mentioned in the text.
2. **Ambiguous, Vague, or Contradictory Data:**
   - **Date of diagnosis:** The text states "March 2023." **Action Taken:** Per the rule, the 1st of the month (2023-03-01) was used.
   - **Timeline Errors:** The date of 1L treatment initiation ("March 25, 2022") is listed as being one year before the date of diagnosis ("March 2023"). This is a logical contradiction. **Action Taken:** The year for the treatment start date was assumed to be a typo and corrected to 2023-03-25 to align with the diagnosis date.

**SYN005 (Baker, William "Bill")**

1. **Ambiguous, Vague, or Contradictory Data:**
   - **Date of diagnosis:** The text states "November 2021." **Action Taken:** Per the rule, the 1st of the month (2021-11-01) was used.
   - **Timeline Errors:** The 1L therapy start date is written as "Dec 9, 20221," which is a typographical error. **Action Taken:** The date was corrected to 2021-12-09 based on the clinical context.

**SYN006 (Jimenez, Maria)**

1. **Missing Data Points:**
   - **Site of distant metastasis at first diagnosis:** The text confirms Stage IV diagnosis but does not specify the location of the metastases at that time.
2. **Ambiguous, Vague, or Contradictory Data:**
   - **Gender:** The text identifies the patient as "Mrs. Jimenez" (female) but also refers to her as a "64-year-old male." **Action Taken:** Gender was recorded as 'f' based on the name and title.

**SYN007 (XXX, Diana)**

1. **Missing Data Points:**
   - **Name:** The patient's full name is redacted ("Diana XXX").
2. **Ambiguous, Vague, or Contradictory Data:**
   - **Driver mutation:** The "Primary Diagnosis" section states "no driver mutations," but the detailed "Comprehensive NGS Panel" lists "ALK: Positive for rearrangement." This is a direct contradiction. **Action Taken:** The specific NGS panel result (ALK fusion) was prioritized over the summary statement.

**SYN008 (Rahman, XXX)**

1. **Missing Data Points:**
   - **Name:** The patient's first name is not provided ("Ms. Rahman"). The name in the demographics block ("Dr. Maxwell Chen") appears to be the Primary Care Provider, not the patient.
   - **PD-L1 status:** Not mentioned in the text.

**SYN009 (Davies, Michael John)**

1. **Missing Data Points:**
   - **1L treatment:** The specific MET inhibitor (TKI) used is not named. It could be Capmatinib or Tepotinib, but this cannot be confirmed.
2. **Ambiguous, Vague, or Contradictory Data:**
   - **Date of diagnosis:** The text states "October 2022." **Action Taken:** Per the rule, the 1st of the month (2022-10-01) was used.
   - **Timeline Errors:** The Date of Birth is listed as "05/25/1997," which would make the patient 26 years old at the time of consultation. However, the text describes him as a "66-year-old gentleman." This is a significant contradiction. **Action Taken:** The year of birth was estimated as 1957 based on the stated age.

**SYN010 (Chen, Sophia)**

1. **Missing Data Points:**
   - **Date of initiation of 1L treatment:** The exact start date for Dabrafenib+Trametinib is not provided.
   - **Assumptions necessary for PFS/OS:** The start date was estimated as 2023-01-30, shortly after the diagnosis on 2023-01-15. This assumption directly impacts the PFS and OS calculations.
2. **Ambiguous, Vague, or Contradictory Data:**
   - **Site of distant metastasis at first diagnosis:** The text states "hepatic metastases (right femur, T5, T9, L2 vertebrae, right scapula)." "Hepatic" refers to the liver, while the listed locations are bones. This is contradictory. **Action Taken:** It was assumed "hepatic" was a typo for "osseous," and the site was recorded as Bone.
   - **Driver mutation:** The text lists "BARF V600E mutation," which is a typographical error. **Action Taken:** Corrected to BRAF V600E.
   - **Date of progression on 1L treatment:** The text states "December 2023." **Action Taken:** Per the rule, the 1st of the month (2023-12-01) was used.

**SYN011 (Moletti, Tobias)**

1. **Missing Data Points:**
   - **Driver mutation:** Molecular testing results for driver mutations are not mentioned. **Action Taken:** Assumed to be Wild-Type (WT).
2. **Ambiguous, Vague, or Contradictory Data:**
   - **Date of initiation of 1L treatment:** The text states "July 2021." **Action Taken:** Per the rule, the 1st of the month (2021-07-01) was used.
   - **Date of progression on 1L treatment:** The exact date is not provided. The text states 1L therapy ran until "December 2022." **Action Taken:** The date was assumed to be 2022-12-30 for calculation purposes.

**SYN012 (Vance, Eleanor Grace)**

1. **Missing Data Points:**
   - **Date of birth:** Not mentioned in the text.
2. **Ambiguous, Vague, or Contradictory Data:**
   - **Site of distant metastasis at first diagnosis:** The text states "No distant mets identified" but also gives a diagnosis of "Stage IV Lung Adenocarcinoma" based on pleural effusion. Malignant pleural effusion (M1a) is a criterion for Stage IV. This is a semantic contradiction. **Action Taken:** The site was correctly identified as Pleura based on the clinical findings.
   - **1L treatment:** The text lists "Caboplatin," which is a typographical error. **Action Taken:** Corrected to Carboplatin.

**SYN013 (Webster, Thomas)**

1. **Missing Data Points:**
   - **1L treatment:** The specific first-line therapy regimen is not named in the text.
2. **Ambiguous, Vague, or Contradictory Data:**
   - **Timeline Errors:** The note, completed on 2025-04-13, states the patient "died peacefully on April 14, 2024." This date of death is before the date the note was written. **Action Taken:** The year of death was assumed to be a typo and corrected to 2025-04-14.

**SYN014 (Rossi, Isabella Francesca)**

- No issues identified.

**SYN015 (Harrison, George Franklin)**

1. **Missing Data Points:**
   - **PD-L1 status:** Not mentioned in the text.
   - **Date of initiation of 1L treatment:** The exact start date is not provided.
   - **Date of progression on 1L treatment:** The exact date is not provided.
   - **Assumptions necessary for PFS/OS:** The 1L start date was estimated as 2021-04-15 (following a March 2021 diagnosis) and the progression date was estimated as 2021-11-15 (prior to the December 2021 start of 2L therapy). These assumptions directly impact the PFS and OS calculations.

**SYN016 (XXX)**

1. **Missing Data Points:**
   - **Name:** The patient's name is not provided.
2. **Ambiguous, Vague, or Contradictory Data:**
   - **Date of diagnosis:** The text states "Jan 2022." **Action Taken:** Per the rule, the 1st of the month (2022-01-01) was used.
   - **Driver mutation:** The text states "KRAS G12C mutation identified. EGFR/ALK/ROS1/BRAF pos." This is contradictory, as a KRAS mutation would typically mean the others are negative (wild-type). "pos." is likely a typo for "neg." **Action Taken:** KRAS G12C was recorded as the driver mutation.

**SYN017 (Thompson, Harold)**

1. **Missing Data Points:**
   - **1L treatment:** The specific first-line TKI used is not named.
2. **Ambiguous, Vague, or Contradictory Data:**
   - **Date of birth:** The DOB is written as "9/17/4," which is incomplete. **Action Taken:** The year was determined to be 1944 based on the patient's stated age of 76 in September 2020.
   - **Date of diagnosis:** The text states "September 2020." **Action Taken:** Per the rule, the 1st of the month (2020-09-01) was used.

**SYN019 (Wilson, Oliver)**

1. **Missing Data Points:**
   - **Date of birth:** Not mentioned in the text.
2. **Ambiguous, Vague, or Contradictory Data:**
   - **Date of diagnosis:** The text states "Jarnury 2021," a typo for January. **Action Taken:** Per the rule, the 1st of the month (2021-01-01) was used.
   - **PD-L1 status:** The text is contradictory, stating "low" expression but also "TPS 0100%". "0100%" is an invalid number. Given the "low" descriptor and CPS of 5, it is highly likely a typo. **Action Taken:** The status was categorized as '<1%'.

**SYN020 (Donovan, Robert)**

1. **Missing Data Points:**
   - **Driver mutation:** Not mentioned in the text. **Action Taken:** Assumed to be Wild-Type (WT).
2. **Ambiguous, Vague, or Contradictory Data:**
   - **Timeline Errors:** The date of diagnosis is listed as "December 3, 2009," but the entire treatment timeline begins in December 2019. **Action Taken:** The year was assumed to be a typo and corrected to 2019-12-03.

**SYN021 (Abernathy, Thomas Frederick)**

1. **Missing Data Points:**
   - **1L treatment:** The specific first-line therapy regimen is not named in the text.

**SYN022 (Rodriguez, Maria Isabel)**

1. **Missing Data Points:**
   - **PD-L1 status:** Not mentioned in the text.
2. **Ambiguous, Vague, or Contradictory Data:**
   - **Date of diagnosis:** The text states "March 2022." **Action Taken:** Per the rule, the 1st of the month (2022-03-01) was used.

**SYN023 (Finch, Harold Edgar)**

1. **Missing Data Points:**
   - **Date of diagnosis:** Not explicitly stated. **Action Taken:** Estimated as 2021-08-15, prior to the 1L start date of 2021-09-10.
2. **Ambiguous, Vague, or Contradictory Data:**
   - **1L treatment:** The regimen is listed as "Carbo/Pembro/Pembrolizumab," which is redundant and not a standard name. **Action Taken:** Assumed to be a typo for the standard Carboplatin/Pemetrexed/Pembrolizumab (Carbo/Pem/Pembro).
   - **Date of progression on 1L treatment:** The text states progression occurred after "~9 months" and treatment ended in "June 2022." **Action Taken:** The date was estimated as 2022-06-15 for calculation purposes.

**SYN024 (Cruz, Josephine)**

1. **Missing Data Points:**
   - **Date of last contact:** The note itself is undated. **Action Taken:** The date of the most recent imaging (2023-08-10) was used as the last known date for PFS/OS calculation.
2. **Ambiguous, Vague, or Contradictory Data:**
   - **1L treatment:** The "Active Problem" lists Entrectinib, but the "Pertinent Oncologic History" and "Medications" sections list Larotrectinib. **Action Taken:** Larotrectinib was used as it was mentioned more frequently and in more detail.

**SYN025 (Jones, Samuel David)**

1. **Missing Data Points:**
   - **Date of birth:** Not mentioned in the text.
2. **Ambiguous, Vague, or Contradictory Data:**
   - **Timeline Errors:** The date of progression is listed as "June 28, 2012," which is eight years before the diagnosis date of July 2020. **Action Taken:** The year was assumed to be a typo and corrected to 2021-06-28.

**SYN026 (Johnson, Sarah)**

1. **Missing Data Points:**
   - **Date of diagnosis:** Not explicitly stated. **Action Taken:** Estimated as 2021-04-15, prior to the 1L start date in May 2021.
2. **Ambiguous, Vague, or Contradictory Data:**
   - **1L treatment start:** The text states "May 2021." **Action Taken:** Per the rule, the 1st of the month (2021-05-01) was used.
   - **1L treatment:** The text lists "alctinib," which is a typographical error. **Action Taken:** Corrected to Alectinib.

**SYN027 (XXX, Patricia Ann)**

1. **Missing Data Points:**
   - **Name:** The patient's full name is redacted ("Patricia Ann ('Patty') XXX").
2. **Ambiguous, Vague, or Contradictory Data:**
   - **Driver mutation:** The text lists "TW," which is not a standard abbreviation. **Action Taken:** Assumed to mean "Tumor Wild-type" and recorded as WT.
   - **Date of diagnosis:** The text states "Oct 2020." **Action Taken:** Per the rule, the 1st of the month (2020-10-01) was used.
   - **Date of initiation of 1L treatment / progression:** The text gives date ranges ("Oct 2020 - Aug 2022"). **Action Taken:** Per the rule, mid-month dates (2020-10-15 and 2022-08-15) were assumed for calculations.

**SYN028 (Parker, Emily)**

1. **Missing Data Points:**
   - **PD-L1 status:** Not mentioned in the text.
2. **Ambiguous, Vague, or Contradictory Data:**
   - **Timeline Errors:** The date of 1L treatment initiation ("June 29, 2023") is listed as being one year before the date of diagnosis ("June 7, 2024"). This is a logical contradiction. **Action Taken:** The year for the treatment start date was assumed to be a typo and corrected to 2024-06-29 to align with the diagnosis date.

**SYN030 (Davis, Katherine)**

1. **Missing Data Points:**
   - **Date of birth:** Not mentioned in the text.

**SYN031 (Davis, Robert Charles)**

1. **Missing Data Points:**
   - **Driver mutation:** Not mentioned in the text. This is a significant logical inconsistency, as the patient was treated with Osimertinib, a therapy specifically for EGFR-mutated cancer. **Action Taken:** The field is marked 'n/a' due to the lack of explicit information.
2. **Ambiguous, Vague, or Contradictory Data:**
   - **Date of diagnosis:** The text states "May 2022." **Action Taken:** Per the rule, the 1st of the month (2022-05-01) was used.
   - **Timeline Errors:** The 1L treatment start date is listed as "June 2, 2023," over a year after the May 2022 diagnosis. This is an unusually long delay. **Action Taken:** The year was assumed to be a typo and corrected to 2022-06-02.

**SYN032 (Thompson, Ronald)**

1. **Missing Data Points:**
   - **Driver mutation:** Not mentioned in the text. **Action Taken:** Assumed to be Wild-Type (WT).
2. **Ambiguous, Vague, or Contradictory Data:**
   - **PD-L1 status:** The text is contradictory, stating "TPS ≥50%" but providing an "actual score 5%." **Action Taken:** The specific quantitative value (5%) was used, categorizing it as '1-49%'.

**SYN033 (Chen, Li)**

1. **Missing Data Points:**
   - **Date of last contact:** The "Date of Visit" is redacted ("XXX"). **Action Taken:** The date was estimated as 2023-09-27, three weeks after the known start date of Cycle 1, for PFS/OS calculation.
2. **Ambiguous, Vague, or Contradictory Data:**
   - **Site of distant metastasis at first diagnosis:** The text states "No distant metastases identified" but also gives a diagnosis of "Stage IV" based on malignant pleural effusion. This is a semantic contradiction. **Action Taken:** The site was correctly identified as Pleura.

**SYN034 (Rigby, Eleanor)**

1. **Missing Data Points:**
   - **Date of birth:** Not mentioned in the text.
2. **Ambiguous, Vague, or Contradictory Data:**
   - **Date of diagnosis:** The text states "Feb 2021." **Action Taken:** Per the rule, the 1st of the month (2021-02-01) was used.
   - **Driver mutation:** The text lists "AKL fusion," which is a typographical error. **Action Taken:** Corrected to ALK fusion.

**SYN035 (XXX)**

1. **Missing Data Points:**
   - **Name:** The patient's name is not provided.
2. **Ambiguous, Vague, or Contradictory Data:**
   - **Driver mutation:** The text is contradictory, stating "Wild-Type for common drivers" but then "(positive for ROS1-rearrangement)." **Action Taken:** The specific positive finding (ROS1 fusion) was prioritized.

**SYN036 (Robards, Clara Eugenie)**

1. **Missing Data Points:**
   - **PD-L1 status:** Not mentioned in the text.
2. **Ambiguous, Vague, or Contradictory Data:**
   - **Driver mutation:** The text lists "ORS1 fusion," which is a typographical error. **Action Taken:** Corrected to ROS1 fusion.

**SYN037 (Whitmore, Marcus Alan)**

1. **Missing Data Points:**
   - **1L treatment:** The regimen "Cisplatin + Pemetrexed + Pembrolizumab" is not one of the predefined options.
2. **Ambiguous, Vague, or Contradictory Data:**
   - **Site of distant metastasis at first diagnosis:** Not explicitly stated. **Action Taken:** Inferred as Bone and Brain based on presenting symptoms (hip pain, confusion) and subsequent treatment (WBRT).
   - **Date of progression on 1L treatment:** The text states "October 220," a typo. **Action Taken:** Corrected to October 2020 and the 1st of the month (2020-10-01) was used.

**SYN038 (Kessler, Fiona M.)**

- No issues identified.

**SYN039 (Anderson, Barbara Louise)**

1. **Missing Data Points:**
   - **Date of birth:** Not mentioned in the text.
2. **Ambiguous, Vague, or Contradictory Data:**
   - **1L treatment start:** The text states "My 10, 2023," a typo. **Action Taken:** Corrected to May 10, 2023.

**SYN040 (Mancini, Theresa Yvonne)**

1. **Missing Data Points:**
   - **Driver mutation:** Not mentioned in the text. **Action Taken:** Assumed to be Wild-Type (WT).
2. **Ambiguous, Vague, or Contradictory Data:**
   - **Name:** The patient information block lists "Mancini, Theresa Yvonne," but the clinical course refers to "Ms. Graham." **Action Taken:** The name from the patient information block (Mancini) was used.

**A2.11 Example prompt for AML multiple parameter extraction**

You are an expert at extracting medical information from text.

Create a table with the following parameters for all 80 patients:

- ID (or MRN)

- Name (Surname, First name(s))

- Date of birth (YYYY-MM-DD)

- Gender (m/f)

- Date of diagnosis (YYYY-MM-DD)

- ELN 2022 classification

- Mutations

- allogeneic SCT (planned, done, no)

- Date of initiation of 1L treatment (YYYY-MM-DD)

- 1L treatment

- 1L treatment ongoing? (y=ongoing, n=stopped permanently). Note: consolidation treatment means 1L ongoing.

- progression on 1L treatment (0=no progression, 1=progression). Note: alloSCT after CR does not mean progression).

- Alive (0=alive, 1=dead)

- PFS since start of 1L treatment in months

- OS since start of 1L treatment in months

Use exact dates and make sure to extract the correct date (cross-check with other dates in the letter to make sure you use the correct dd/mm!).

if no exact date provided assume 1st of months (and if early 1st, if mid 15th and if end 30th).

Definition of PFS: time from treatment start to disease progression (irrespective of treatment continuation) or last known time point alive without progression

Definition of OS: time from treatment start to death or last known time point alive

example:

letter for AML248 from 2024-03-25, initiation February 2023, still alive and on treatment without progression

| Patient MRN | 1L treatment ongoing (y/n) | progression on 1L (0=no, 1=yes) | PFS since start of 1L (months) | alive (0=alive, 1=dead) | OS since start of 1L (months) |

| AML248 | y | 0 | 14 | 0 | 14 |

For PFS and OS only use full integer months, i.e. 15 months + 13 days = 15 months and 15 months + 17 days = 16 months

**A2.12 Example prompt for hypothesis generation**

You are an expert at extracting medical information from text.

Task: Create a list of potential new scientific hypotheses about correlations, dependencies, causations etc. from these NSCLC patients (e.g. Hb<10 and survival, BMI and mutation etc.)

You are a clinical data scientist and medical researcher with expertise in oncology and analyzing patient records.

Your task is to meticulously analyze the attached 80 synthetic patient records for Non-Small Cell Lung Cancer (NSCLC).

Step 1: Create a table consisting of all relevant data points (ca. 30) for each patient (demographics, treatment, comorbidities, comedication, lab values such as CRP, LDH etc.)

Step 2: Based exclusively on the information contained within these documents, generate one novel, data-grounded scientific hypothesis.

This hypothesis should explore potential correlations, associations, or trends between different clinical, molecular, demographic, laboratory, and outcome variables present in the data.

IMPORTANT RULE: The number of patients in step 2 MUST be consistent with the information in the table from step 1!

Example for hypothesis:

"Step 2: Novel Scientific Hypothesis

Hypothesis: In patients with KRAS-mutated Stage IV Non-Small Cell Lung Adenocarcinoma treated with an immune checkpoint inhibitor (ICI)-containing regimen, the presence of a concurrent STK11 loss-of-function mutation is associated with a significantly increased risk of developing severe (Grade 3) immune-mediated colitis.

Data Grounding and Rationale (Based exclusively on the provided records):

1. Cohort Identification: Within the 80 patient records, a total of 12 patients were identified with a KRAS mutation (subtypes G12V, G12C, or G12D) who received an ICI-based therapy (pembrolizumab, either as monotherapy or in combination with chemotherapy).

2. Sub-Group Analysis: This cohort was stratified based on the presence or absence of a documented concurrent STK11 mutation:

o KRAS/STK11 Co-mutated Group (n=3): Patients SYN002, SYN029, and SYN048 were documented as having both a KRAS and an STK11 mutation.

o KRAS-mutated / STK11 Wild-Type (or not reported) Group (n=9): Patients SYN013, SYN016, SYN022, SYN033, SYN042, SYN049, SYN062, SYN066, and SYN073 had a KRAS mutation without a documented STK11 co-mutation.

3. Observation of Adverse Events: A striking pattern of severe gastrointestinal toxicity emerged when comparing these two groups:

o In the KRAS/STK11 co-mutated group, 2 out of 3 patients (67%) developed Grade 3 immune-mediated colitis. Specifically, patient SYN002 (KRAS G12V/STK11) and patient SYN048 (KRAS G12D/STK11) both experienced this severe adverse event, with patient SYN048 requiring permanent discontinuation of pembrolizumab.

o In contrast, among the 9 patients with a KRAS mutation but no documented STK11 co-mutation, 0 patients (0%) were reported to have developed immune-mediated colitis of any grade.

Conclusion: This analysis suggests that while STK11 co-mutation is often linked to primary resistance to ICIs, it may also predispose KRAS-mutant NSCLC patients to a specific and severe pattern of gastrointestinal immune-related toxicity. This observation warrants further investigation to understand the underlying biological mechanism and to potentially inform patient monitoring and management strategies in this molecularly defined subgroup."

**A2.13 Example letter 1 (SYN007)**

**MOUNTAIN VIEW MEDICAL GROUP**

**ONCOLOGY DEPARTMENT**

**COMPREHENSIVE PATIENT CARE DOCUMENTATION**

**DATE OF SERVICE:** April 13, 2025 **VISIT TYPE:** Scheduled Follow-up & Treatment Administration

**PATIENT INFORMATION**

**Name:** Diana Parker **DOB:** 10/07/1961 **MRN:** SYN007 **Primary Insurance:** United Healthcare (Group #: UH95372) **Secondary Insurance:** AFLAC Cancer Policy **Primary Care Provider:** Dr. Robert Anderson **Emergency Contact:** Michael Parker (husband) - (555) 278-6491

**ONCOLOGIC DISEASE PROFILE**

**Primary Diagnosis:** Stage IV non-small cell lung cancer (adenocarcinoma) **Date of Initial Diagnosis:** June 18, 2022 **Pathologic Details:** Moderately differentiated adenocarcinoma with predominant acinar pattern (70%) and papillary pattern (30%) **Disease Sites:** Primary right upper lobe (1.7cm), liver metastases (multiple, largest 2.1cm), left adrenal metastasis (2.5cm), bone metastases (T7, L3, right iliac wing) **Current Disease Status:** Stable disease with continued response to immunotherapy

**Molecular/Biomarker Profile:**

- **Comprehensive NGS Panel (Foundation One CDx):**
  - EGFR: Negative for activating mutations and resistance mutations
  - ALK: Negative for rearrangements
  - ROS1: Negative for rearrangements
  - BRAF: Negative for V600E and other mutations
  - RET: Negative for fusions
  - MET: Negative for exon 14 skipping and amplification
  - NTRK: Negative for fusions
  - KRAS: Wild-type (no G12C or other mutations)
  - TP53: R273H mutation (pathogenic)
  - STK11: Wild-type
  - KEAP1: Wild-type
  - NF1: Truncating mutation (likely pathogenic)
  - ATM: Variant of uncertain significance
  - CDKN2A/B: Homozygous deletion
  - PD-L1 (22C3 pharmDx): 90% TPS (high expresser, ≥50% cutoff)
  - Tumor Mutational Burden: 12 mutations/Mb (intermediate)
  - Microsatellite Status: Stable (MSS)
  - HLA typing: A*02:01 positive (potentially relevant for future immunotherapy trials)

**TREATMENT HISTORY**

**First-line Therapy:** Pembrolizumab 200mg IV q3weeks **Start Date:** July 10, 2022 **Current Cycle:** #46 **Best Response:** Partial response (RECIST 1.1) **Recent Response Assessment:** Continued partial response with further decrease in target lesions

**Detailed Treatment Timeline:**

- **Diagnosis Workup (June 2022):**
  - Core needle biopsy of liver lesion confirmed metastatic lung adenocarcinoma
  - PET/CT showed hypermetabolic primary RUL lesion (SUV 14.6), multiple liver metastases (SUV 8.2-11.3), left adrenal mass (SUV 7.8), and bone metastases (SUV 5.4-8.7)
  - Brain MRI negative for intracranial metastases
  - Molecular testing completed as detailed above
- **Initial Treatment Phase (July 2022 - December 2022):**
  - Pembrolizumab 200mg IV q3weeks initiated July 10, 2022
  - First response assessment (September 2022): Partial response with 38% reduction in target lesions
  - Palliative radiation to symptomatic T7 metastasis (30 Gy in 10 fractions, completed August 2022)
  - Zoledronic acid initiated for bone metastases (August 2022)
- **Continued Treatment Phase (January 2023 - Present):**
  - Ongoing pembrolizumab with sustained disease control
  - Development of immune-related hypothyroidism (January 2024) - grade 1, managed with levothyroxine
  - Transient immune-related hepatitis (grade 2, July 2024) requiring 4-week treatment hold and low-dose prednisone, with complete resolution and successful rechallenge
  - Most recent imaging (April 2025) shows continued partial response

**Significant Adverse Events:**

- Grade 1 hypothyroidism (managed with levothyroxine)
- Grade 2 immune-mediated hepatitis (resolved)
- Grade 2 fatigue (intermittent)
- Grade 1 pruritus (managed with topicals)
- Grade 1 arthralgias (managed with acetaminophen)

**Supportive Care Interventions:**

- Zoledronic acid 4mg IV q3months for bone metastases (initiated August 2022)
- Calcium and vitamin D supplementation
- Physical therapy for strengthening after initial diagnosis
- Nutritional counseling
- Cancer support group participation
- Mindfulness-based stress reduction program completion

**COMORBID CONDITIONS**

- Essential hypertension (diagnosed 2010)
- Hyperlipidemia (diagnosed 2015)
- Osteopenia (diagnosed 2019)
- Generalized anxiety disorder (well-controlled)
- History of migraines (rare since cancer diagnosis)
- Status post cholecystectomy (2008)
- Status post total hysterectomy for fibroids (2005)

**COMPREHENSIVE CURRENT VISIT DOCUMENTATION**

**Chief Concern:** Scheduled follow-up and cycle 46 of pembrolizumab

**Interval History:** Mrs. Parker presents for routine 3-week follow-up and cycle 46 of pembrolizumab immunotherapy. Since her last visit, she reports stable energy levels with mild fatigue typically occurring 2-3 days post-infusion but resolving spontaneously. She describes her overall quality of life as "good" and continues to engage in most of her usual activities.

She maintains an active lifestyle with daily walks of approximately 1 mile without limitation. She reports no bone pain or discomfort at known sites of skeletal metastases. No new sites of pain have developed. Her appetite remains good with stable body weight.

Patient reports occasional mild arthralgias affecting knees and wrists, primarily in the morning and relieved with stretching exercises and occasional acetaminophen. She denies any rash, pruritus, cough, dyspnea, chest pain, abdominal pain, diarrhea, or other concerning symptoms that might suggest progression or treatment-related toxicity.

Mrs. Parker continues to work part-time (3 days weekly) as an interior designer. She notes that returning to work has been important for her emotional well-being and sense of normalcy. She engages in social activities regularly and has recently started volunteering at a local cancer support organization, providing peer mentorship to newly diagnosed patients.

She adheres to regular sleep schedule and follows a Mediterranean diet as recommended by nutritional services. She has maintained her exercise regimen of daily walking and twice-weekly light resistance training. No falls or injuries since last visit.

**Current Medications:**

1. Pembrolizumab 200mg IV q3weeks
2. Levothyroxine 50mcg PO daily
3. Amlodipine 5mg PO daily
4. Rosuvastatin 10mg PO daily
5. Vitamin D3 2000 IU PO daily
6. Calcium carbonate 600mg PO BID
7. Escitalopram 10mg PO daily
8. Acetaminophen 650mg PO PRN joint pain/headache
9. Multivitamin 1 tablet PO daily
10. Melatonin 3mg PO qhs PRN insomnia

**Allergies:**

- Penicillin (hives)
- Sulfa drugs (rash)
- Contrast dye (nausea, flushing)

**Social History:**

- Married 40 years to husband Michael, who attends appointments
- Two adult children (ages 38, 35) living nearby with four grandchildren
- Former smoker (15 pack-years, quit 1995)
- Alcohol: Occasional glass of wine (1-2/week)
- No recreational drug use
- Lives in two-story home with husband (bedroom on main floor)
- Recently retired from full-time work but maintains part-time interior design consulting
- Exercises regularly (daily walking, light resistance training)
- Active in church community and cancer support network

**Family History:**

- Father: Died age 78, myocardial infarction
- Mother: Died age 82, complications of Alzheimer's disease
- Sister: Alive, age 66, breast cancer survivor (diagnosed age 52)
- Brother: Alive, age 58, hypertension
- No family history of lung cancer

**Review of Systems:** CONSTITUTIONAL: Reports mild fatigue 2-3 days post-treatment, otherwise denies fever, chills, night sweats, significant weight changes. HEENT: Denies headache, visual changes, hearing changes, tinnitus, sinus congestion, oral lesions. RESPIRATORY: Denies cough, hemoptysis, wheezing, shortness of breath at rest or with normal activities. CARDIOVASCULAR: Denies chest pain, palpitations, orthopnea, PND, peripheral edema. GASTROINTESTINAL: Appetite good, denies nausea, vomiting, diarrhea, constipation, abdominal pain, changes in bowel habits. GENITOURINARY: Denies dysuria, frequency, urgency, hematuria, incontinence. MUSCULOSKELETAL: Reports occasional mild morning arthralgias in knees and wrists. Denies bone pain at known metastatic sites. SKIN: Denies rash, pruritus, unusual dryness, color changes, lesions. NEUROLOGICAL: Denies headache, dizziness, syncope, weakness, sensory changes, balance problems. PSYCHIATRIC: Denies depression, significant anxiety, mood disturbances, sleep difficulties. ENDOCRINE: Denies polyuria, polydipsia, heat or cold intolerance. HEMATOLOGIC/LYMPHATIC: Denies easy bruising, bleeding, enlarged lymph nodes. IMMUNOLOGIC: Denies recurrent infections, unusual allergic reactions.

**Physical Examination:** VITALS: Temperature 98.2°F, Heart Rate 68 (regular), Respiratory Rate 16, Blood Pressure 126/72 mmHg, Oxygen Saturation 97% on room air, Weight 148 lbs (stable, BMI 25.4)

GENERAL: Well-appearing woman in no acute distress. Alert, oriented, and engaging. Well-groomed with good hygiene.

HEENT: Normocephalic, atraumatic. Pupils equal, round, reactive to light and accommodation. Extraocular movements intact in all directions. Visual fields full to confrontation. Sclerae anicteric, conjunctivae pink. Oropharynx moist, no lesions, no thrush. No cervical lymphadenopathy.

NECK: Supple, no lymphadenopathy, thyroid normal in size without nodules.

LUNGS: Clear to auscultation bilaterally. No wheezes, rhonchi, or crackles. Normal respiratory effort. No chest wall tenderness.

HEART: Regular rate and rhythm. Normal S1 and S2. No murmurs, gallops, or rubs. No displacement of point of maximal impulse.

ABDOMEN: Soft, non-tender, non-distended. No hepatosplenomegaly. Normal bowel sounds. No masses or bruits. Surgical scars from prior cholecystectomy well-healed.

EXTREMITIES: No clubbing, cyanosis, or edema. Full range of motion of all joints without significant pain or limitation. No joint swelling, erythema, or deformity. Normal muscle tone and strength 5/5 throughout.

SKIN: Warm, dry, normal turgor. No rashes, lesions, or unusual pigmentation. No palmar erythema or spider angiomata.

LYMPHATIC: No cervical, supraclavicular, axillary, or inguinal lymphadenopathy.

NEUROLOGICAL: Alert and oriented to person, place, time, and situation. Cranial nerves II-XII intact. Motor strength 5/5 in all extremities. Sensation intact to light touch, pin, temperature in all extremities. Reflexes 2+ and symmetric. Coordination normal with finger-to-nose and heel-to-shin testing. Gait steady and normal, Romberg negative.

PSYCHIATRIC: Pleasant and cooperative. Appropriate affect. Normal thought process and content. No evidence of depression or anxiety.

**Laboratory Findings (04/13/2025):** CBC:

- WBC: 5.2 K/uL (normal: 4.0-11.0)
- Hemoglobin: 12.1 g/dL (slightly low, normal: 12.5-15.5)
- Hematocrit: 36.3% (slightly low, normal: 36-46%)
- Platelets: 189 K/uL (normal: 150-400)
- Absolute Neutrophil Count: 3.1 K/uL (normal: 1.8-7.7)
- Absolute Lymphocyte Count: 1.4 K/uL (normal: 1.0-4.8)

Comprehensive Metabolic Panel:

- Sodium: 138 mEq/L (normal: 136-145)
- Potassium: 4.2 mEq/L (normal: 3.5-5.1)
- Chloride: 104 mEq/L (normal: 98-107)
- CO2: 25 mEq/L (normal: 22-29)
- BUN: 14 mg/dL (normal: 7-20)
- Creatinine: 0.9 mg/dL (normal: 0.6-1.1)
- Glucose: 92 mg/dL (normal: 70-99)
- Calcium: 9.4 mg/dL (normal: 8.5-10.2)
- Phosphorus: 3.6 mg/dL (normal: 2.5-4.5)
- Magnesium: 2.0 mg/dL (normal: 1.7-2.2)
- AST: 28 U/L (normal: 10-40)
- ALT: 32 U/L (normal: 7-56)
- Alkaline Phosphatase: 86 U/L (normal: 40-129)
- Total Bilirubin: 0.8 mg/dL (normal: 0.1-1.2)
- Albumin: 4.1 g/dL (normal: 3.4-5.0)
- Total Protein: 7.2 g/dL (normal: 6.4-8.2)
- LDH: 168 U/L (normal: 140-271)

Thyroid Function:

- TSH: 3.2 mIU/L (normal: 0.4-4.5)
- Free T4: 1.1 ng/dL (normal: 0.8-1.8)

Immune Monitoring:

- ANA: Negative
- Rheumatoid Factor: Negative
- C-Reactive Protein: 0.8 mg/dL (normal: <1.0)
- ESR: 18 mm/hr (normal for age: <30)

Tumor Markers:

- CEA: 2.4 ng/mL (normal: <3.0, decreased from 18.6 ng/mL at diagnosis)
- CYFRA 21-1: 1.8 ng/mL (normal: <3.3, decreased from 8.4 ng/mL at diagnosis)

**Imaging Studies:**

**CT Chest/Abdomen/Pelvis (04/02/2025):** TECHNIQUE: Multidetector CT of the chest, abdomen, and pelvis was performed after administration of intravenous contrast. Oral contrast was also administered. Images were reconstructed in axial, coronal, and sagittal planes.

FINDINGS: CHEST:

- Right upper lobe nodule measures 1.2 cm (decreased from 1.8 cm on previous study from 01/05/2025 and from original 4.3 cm at baseline).
- No hilar or mediastinal lymphadenopathy.
- No pleural effusion or pneumothorax.
- No evidence of pulmonary embolism.
- Coronary and aortic calcifications noted, unchanged.

ABDOMEN:

- Liver: Previously noted multiple hepatic lesions have decreased in size and number. Currently three identifiable hypodense lesions, largest measuring 1.2 cm in segment VII (previously 2.1 cm). No new hepatic lesions.
- Left adrenal mass measures 1.8 cm (decreased from 2.5 cm at baseline).
- Spleen, pancreas, and right adrenal gland appear normal.
- No lymphadenopathy.
- Kidneys normal in size and enhancement without hydronephrosis or stones.
- Status post cholecystectomy.
- Status post hysterectomy.

PELVIS:

- No pelvic masses or lymphadenopathy.
- No free fluid.
- Sclerotic focus in right iliac wing, unchanged, consistent with treated metastasis.

BONES:

- Sclerotic changes in T7 and L3 vertebral bodies, unchanged, consistent with treated bone metastases.
- No new osseous lesions identified.

IMPRESSION:

1. Continued partial response to therapy with further 33% decrease in size of primary right upper lobe nodule since January 2025.
2. Decreased size and number of hepatic metastases.
3. Stable left adrenal metastasis.
4. Stable sclerotic changes in known bone metastases without evidence of new bone lesions.
5. No evidence of disease progression.

**Brain MRI with contrast (04/02/2025):** TECHNIQUE: Multiplanar multisequence MRI of the brain was performed before and after administration of gadolinium contrast.

FINDINGS:

- No evidence of intracranial metastases.
- No abnormal enhancement.
- No parenchymal lesions.
- Ventricles and sulci normal in size and configuration.
- No midline shift or mass effect.
- No abnormal signal in the brainstem or cerebellum.
- No acute infarction, hemorrhage, or white matter disease.

IMPRESSION: No evidence of intracranial metastases or other abnormalities.

**Recent Echocardiogram (03/15/2025):**

- Normal left ventricular size and function
- Left ventricular ejection fraction 60-65%
- Normal right ventricular size and function
- No significant valvular abnormalities
- No pericardial effusion

**Pulmonary Function Tests (03/15/2025):**

- FEV1: 2.48 L (92% predicted)
- FVC: 3.21 L (95% predicted)
- FEV1/FVC ratio: 77%
- DLCO: 18.2 mL/mmHg/min (85% predicted)
- Interpretation: Normal spirometry and diffusion capacity

**ASSESSMENT AND PLAN**

**Assessment:** Mrs. Diana Parker is a 63-year-old female with stage IV NSCLC (adenocarcinoma), PD-L1-high (90% TPS), wild-type for all targetable driver mutations, with metastases to liver, adrenal gland, and bone at diagnosis. She has demonstrated sustained partial response to pembrolizumab monotherapy.

Current disease status shows ongoing partial response with decreasing size of primary tumor (now 1.2 cm, reduced from 4.3 cm at baseline), significant reduction in hepatic metastases (largest now 1.2 cm, reduced from 2.1 cm at baseline), stable adrenal metastasis, and stable sclerotic changes in bone metastases. No new sites of metastatic disease have developed during treatment course.

Patient continues to tolerate therapy well with only mild immune-related hypothyroidism requiring thyroid hormone replacement. Previous episode of grade 2 immune-mediated hepatitis (July 2024) resolved completely with temporary treatment hold and low-dose corticosteroids. She has maintained excellent performance status (ECOG 0-1) throughout treatment and reports good quality of life with minimal treatment-related side effects.

**Treatment Plan:**

1. Continue pembrolizumab 200mg IV today (Cycle 46)
2. Standard premedications with diphenhydramine 25mg IV and famotidine 20mg IV administered
3. Continue vitamin D3 2000 IU PO daily and calcium carbonate 600mg PO BID
4. Continue levothyroxine 50mcg PO daily for immune-related hypothyroidism
5. Administer zoledronic acid 4mg IV today (q3months for bone metastases)
6. Next imaging: CT chest/abdomen/pelvis in 3 months (July 2025)
7. Brain MRI for surveillance in 3 months (July 2025)
8. Return for next cycle in 3 weeks (05/04/2025)
9. Next thyroid function panel in 6 weeks

**Duration of Therapy Discussion:** We had an extensive discussion regarding the optimal duration of immunotherapy treatment. Mrs. Parker has now completed 46 cycles and would reach the 3-year benchmark in 3 more cycles.

Recent retrospective data and emerging clinical trial results suggest potential for treatment discontinuation after prolonged response without significant impact on long-term outcomes. The STOP-GAP strategy (treatment until maximal response followed by treatment holiday with close monitoring and retreatment upon progression) has shown promising results in selected patients.

Given Mrs. Parker's exceptional response duration, continued response on recent imaging, and stable disease status, we discussed three potential approaches:

1. Complete the standard 2-year course (3 more cycles) and discontinue
2. Continue beyond 2 years given ongoing benefit and excellent tolerance
3. Consider treatment holiday after completing 2 years with close monitoring

After thorough discussion of risks and benefits of each approach, including consideration of potential immune-related adverse events with continued therapy versus risk of progression with discontinuation, Mrs. Parker expressed preference for completing the standard 2-year course (3 more cycles) and then discontinuing with close surveillance. She understands that treatment can be reinitiated if disease progression occurs after discontinuation.

**Immune-related Adverse Events Management:** We reviewed potential signs and symptoms of immune-mediated toxicities affecting various organ systems (colitis, pneumonitis, hepatitis, endocrinopathies, dermatitis, nephritis, neurological events) and instructed patient to report any new or concerning symptoms promptly. Given her prior episode of immune-mediated hepatitis, we will continue to monitor liver function tests with each treatment cycle. Thyroid function will continue to be monitored every 6 weeks during therapy.

**Survivorship Planning:** Mrs. Parker continues to participate in our institution's comprehensive survivorship program. She has completed advance care planning documentation and designated her husband as healthcare power of attorney. Social work continues to assist with insurance navigation and financial planning. Nutrition and exercise consultations have been completed with personalized recommendations, which she reports following consistently.

Her survivorship care plan includes:

1. Maintaining Mediterranean diet pattern
2. Continuing regular physical activity (daily walking, resistance training)
3. Adhering to regular cancer surveillance schedule
4. Participation in cancer support group activities
5. Engaging in volunteer peer mentorship program
6. Annual wellness visits with primary care physician
7. Routine health maintenance including mammography, colonoscopy, bone density, and dental care

**Future Therapeutic Considerations:** While current therapy continues to provide benefit, we discussed potential future treatment options if progression occurs after completion of planned immunotherapy course. These include:

1. Rechallenge with pembrolizumab if progression occurs after significant treatment-free interval
2. Docetaxel ± ramucirumab for second-line therapy
3. Consideration of platinum-based chemotherapy combinations
4. Enrollment in clinical trials based on genomic profiling results
5. Evaluation for additional targetable mutations at progression through repeat biopsy or liquid biopsy
6. Local therapies for oligoprogressive disease if appropriate

**Psychosocial Support:** Patient continues to demonstrate remarkable psychological resilience throughout her treatment journey. She reports effective coping strategies including spiritual practice, family support, creative activities, and participation in support groups. She describes her mood as stable and positive with no significant anxiety or depression symptoms. She has developed meaning and purpose through her volunteer activities, providing peer support to newly diagnosed cancer patients.

She maintains strong social support network including her husband of 40 years, two adult children, and extended family and friends. Her part-time work as an interior designer provides professional fulfillment and sense of normalcy. Overall quality of life is reported as high despite cancer diagnosis and ongoing treatment.

**TREATMENT ADMINISTERED TODAY:**

- Pembrolizumab 200mg IV over 30 minutes
- Premedications: Diphenhydramine 25mg IV, Famotidine 20mg IV
- Zoledronic acid 4mg IV over 15 minutes
- Treatment tolerated without acute complications

**FOLLOW-UP PLAN:**

- Return to clinic in 3 weeks (05/04/2025) for next cycle of pembrolizumab
- Laboratory testing prior to next visit: CBC, CMP, TSH
- Next imaging scheduled for July 2025 (CT chest/abdomen/pelvis, brain MRI)
- Call with any new or worsening symptoms
- Patient provided with after-hours contact information: (555) 789-1234

**CLINICAL TRIALS CONSIDERATION:** Patient was screened for eligibility in protocol INSPIRE-NSCLC-203 (Immunotherapy Continuation vs. Observation After 2 Years of PD-1 Inhibitor Therapy in NSCLC) and would be eligible upon completion of 2 years of pembrolizumab therapy. We provided information about this randomized study, which evaluates continuation of immunotherapy versus observation with the option of retreatment at progression. She expressed interest in learning more about this trial option as she approaches completion of planned therapy duration.

**ADDITIONAL RECOMMENDATIONS:**

1. Continue current supportive care regimen
2. Maintain bone health with calcium, vitamin D, and zoledronic acid
3. Consider cognitive assessment at completion of therapy to establish post-treatment baseline
4. Annual influenza vaccination; pneumococcal vaccination up-to-date
5. Skin examination by dermatology annually
6. Dental evaluation every 6 months while on zoledronic acid

This comprehensive assessment and plan was discussed in detail with Mrs. Parker and her husband. All questions were addressed, and they expressed understanding and agreement with the proposed approach. Patient was given the opportunity to ask questions and expressed satisfaction with the information provided.

Electronically signed by:

Susan Walker, MD Board Certified Medical Oncology Mountain View Medical Group License #: MD12345 NPI: 1234567890 Contact: (555) 789-1234

Reviewed and co-signed by: Jonathan Blackwell, MD, PhD Director, Thoracic Oncology Program

cc: Dr. Robert Anderson (Primary Care) Dr. Elizabeth Chen (Pulmonology) Dr. James Wilson (Cardiology) Dr. Sarah Johnson (Endocrinology)

**A2.14 Example letter 2 (SYN020)**

# TERMINAL CARE REPORT FOR ID SYN020

**DIAGNOSIS**

Metastatic non-small cell lung carcinoma (adenocarcinoma), wild-type, PDL1 TPS <1% (CPS not applicable)

Date of diagnosis: December 3, 2019

Metastatic sites: Adrenal glands (bilateral), Bone (T4, T7, T10 vertebrae, right iliac crest, left femoral head)

**MEDICAL HISTORY**

- COPD (40 pack-year smoking history, quit 2018)

- Coronary artery disease s/p CABG (2015)

- Chronic kidney disease (Stage III)

- Essential hypertension

**ONCOLOGICAL TIMELINE**

- First-line therapy: Carboplatin AUC 5 + Paclitaxel 175 mg/m² (q21d)

* Start date: December 28, 2019

* End date: June 25, 2020

* Best response: Partial response

- Second-line therapy: Docetaxel 75 mg/m² (q21d)

* Start date: July 15, 2020

* End date: October 29, 2020

* Best response: Stable disease

- Third-line therapy: Clinical trial XRT-290 (experimental MEK inhibitor)

* Start date: November 18, 2020

* End date: December 28, 2020

* Reason for discontinuation: Disease progression and declining performance status

- Palliative care referral: January 2, 2021

- Date of death: January 19, 2021

**PATHOLOGY**

Initial biopsy (December 3, 2019):

Core needle biopsy of right upper lobe mass showed moderately differentiated adenocarcinoma of lung origin.

Immunohistochemistry: TTF-1 positive, CK7 positive, CK20 negative, Napsin A positive, p40 negative

Molecular testing:

- EGFR: Wild-type

- ALK: No rearrangement

- ROS1: No rearrangement

- BRAF: Wild-type

- KRAS: Wild-type

- NTRK: No fusions detected

- MET: No exon 14 skipping

- RET: No fusions detected

- PDL1 (22C3): TPS <1% (negative)

**TERMINAL ADMISSION DETAILS**

Admission date: January 10, 2021

Admitted to: St. Mary's Hospital Palliative Care Unit

Primary concern: Severe dyspnea, fatigue, cachexia

ECOG performance status: 4

Management:

- Pain: Morphine PCA pump

- Dyspnea: Supplemental oxygen, morphine, and lorazepam

- Anxiety: Lorazepam and psychological support

- Secretions: Glycopyrrolate

Patient's condition steadily declined with progressive respiratory failure. Per previously established goals of care, comfort measures were prioritized. Patient passed away peacefully on January 19, 2021, at 03:27 with family at bedside.

**FINAL MEDICATIONS**

1. Morphine PCA pump

2. Lorazepam 0.5-1mg IV PRN q4h for anxiety/dyspnea

3. Glycopyrrolate 0.2mg IV q4h PRN for secretions

4. Haloperidol 0.5mg IV PRN q6h for agitation/delirium

**SUMMARY**

Mr. Donovan was a 67-year-old male with metastatic wild-type NSCLC with negative PDL1 expression who progressed through three lines of therapy with an overall survival of 15 months from diagnosis. Terminal care was provided with dignity and comfort measures in accordance with patient and family wishes. Family members were present at time of death and were provided with bereavement support.

Prepared by: Dr. Jessica Langdon, Palliative Care Attending

Date: January 20, 2021

---

**PATIENT INFORMATION**

Name: Robert Donovan

Date of Birth: April 21, 1953

Date of Death: January 19, 2021

Next of kin: Mrs. Janet Donovan (spouse)

Primary Care Physician: Dr. Samuel Watkins

**A2.15 Example letter 3 (SYN092)**

**Patient:** Keith, Catherine Ann **MRN:** SYN092 **DOB:** 08/16/1963
**Date:** Sept 6, 2023
**Provider:** B. Carter, MD

**Dx:** Stage IV Lung Adeno, ALK+ diagnosed 03/03/23, PDL1 10%. Mets to Lung only.
**Rx:** Alectinib 600mg BID since 03/30/23

**S:** Doing very well. asymptomatic. No cough/SOB. Tolerating Alectinib well - mild constipation managed w/ diet/fluids only needed recently. No myalgia/edema/visual sx/rash. ECOG 0.
**O:** Vitals/Exam WNL. Labs (CBC/CMP/LFTs/CPK) WNL. CT Chest (Aug 28, 23): Partial response ongoing, pulm nodules smaller/stable compared to May 23.
**A:** Stable PR to Alectinib, excellent tolerance.
**P:** Cont Alectinib 600mg BID. Labs/F-U in 3 mos. Next CT Chest ~Dec 23. Brain MRI surveillance q6 mos (next ~Oct 23). Cont supportive care PRN constipation. RTC sooner PRN issues.

**BC/onc**

**A2.16 Example letter 4 (SYN117)**

**Patient:** Emilio Williams (* 1952-03-17)
**Medical Record Number:** SYN117
**Admission:** 2025-04-03 - 2025-04-14

**Discharge Diagnosis: Terminal Respiratory Failure Due to Progressive NSCLC After Multiple Lines of Therapy**

**1. Detailed Oncological Diagnosis:**

**Primary Diagnosis:** Non-Small Cell Lung Cancer (NSCLC), Adenocarcinoma, Stage IVA
**Date of Initial Diagnosis:** March 15, 2021

Histology:

- Thoracentesis and pleural biopsy (March 2021) revealed poorly differentiated adenocarcinoma with predominant solid growth pattern.
- Immunohistochemistry: Positive for TTF-1, CK7. Negative for p40, CK20, GATA3.
- Molecular testing: KRAS: G12C mutation positive, EGFR: Wild-type, ALK: No rearrangement, ROS1: No rearrangement, BRAF: Wild-type, MET: No exon 14 skipping mutation, NTRK: No fusion
- PD-L1 expression: <1% Tumor Proportion Score (TPS), CPS 5, IC 2%

Staging:

- TNM (8th edition): cT3N2M1a (Stage IVA)
- Imaging Studies:
  - Chest CT (March 2021): 4.6 cm right lower lobe mass with invasion into visceral pleura, ipsilateral hilar and mediastinal lymphadenopathy, and large right pleural effusion.
  - PET/CT (March 2021): FDG-avid primary mass (SUVmax 14.8), mediastinal lymphadenopathy (stations 4R, 7, 10R), and right pleural effusion/thickening with moderate FDG uptake (SUVmax 6.2).
  - Brain MRI (March 2021): No evidence of brain metastases.

**2. History of Oncological Treatment:**

**First-line Therapy:**

- Carboplatin AUC 5 + Pemetrexed 500 mg/m² + Pembrolizumab 200 mg IV every 3 weeks
- Initiated April 6, 2021
- Completed 4 cycles of triplet therapy, followed by maintenance pemetrexed + pembrolizumab
- Disease progression documented January 14, 2022

**Second-line Therapy:**

- Sotorasib 960 mg PO daily (KRAS G12C inhibitor)
- Initiated February 2022
- Partial response for 5 months
- Disease progression documented July 2022

**Third-line Therapy:**

- Docetaxel 75 mg/m² + Ramucirumab 10 mg/kg IV every 3 weeks
- Initiated August 2022
- Stable disease for 6 months
- Disease progression documented February 2023

**Fourth-line Therapy:**

- Gemcitabine 1000 mg/m² days 1 and 8 of 21-day cycle
- Initiated March 2023
- Stable disease for 3 months, then progression
- Discontinued June 2023

**Fifth-line Therapy:**

- Clinical trial of novel SHP2/KRAS G12C dual inhibitor (Study ID: KR-SHP2-301)
- Initiated July 2023
- Initial stable disease
- Disease progression documented November 2023
- Discontinued from trial December 2023

**Palliative Procedures:**

- Tunneled pleural catheter placement (March 2021)
- Removed after pleurodesis achieved (August 2021)
- Chemical pleurodesis with talc (August 2021)
- Repeat tunneled pleural catheter (January 2024)

**3. Imaging**

- CT Chest/Abdomen/Pelvis (February 2025): Progressive disease with enlarging primary tumor mass (now 6.2 cm), increasing pleural thickening, new small pericardial effusion, and increased mediastinal lymphadenopathy.
- Chest X-ray (April 3, 2025, on admission): Large right pleural effusion with complete opacification of right hemithorax, mediastinal shift to left, bilateral interstitial infiltrates.

**4. Comorbidities:**

- Chronic obstructive pulmonary disease (GOLD stage 3, diagnosed 2014)
- Coronary artery disease s/p MI and PCI (2017)
- Hypertension (diagnosed 2005)
- Type 2 diabetes mellitus (diagnosed 2010)
- Chronic kidney disease stage III (eGFR 45-59)
- Hypothyroidism (diagnosed 2016)
- Peripheral neuropathy (chemotherapy-related)
- History of pulmonary embolism (2022)
- Former smoker (45 pack-year history, quit 2014)

**5. Physical Exam at Admission:**

General: 73-year-old male in moderate respiratory distress, cachectic appearance.

Vitals: BP 135/78 mmHg, HR 102 bpm, RR 28/min, Temp 37.3°C, SpO2 84% on room air, improved to 92% on 4L O₂ via nasal cannula.

HEENT: Normocephalic, atraumatic. Mild conjunctival pallor. No oral lesions.

Neck: Supple. No cervical or supraclavicular lymphadenopathy. No JVD.

Cardiovascular: Tachycardic, regular rhythm. Normal S1, S2. No murmurs, rubs, or gallops.

Respiratory: Absent breath sounds over right hemithorax. Crackles at left base. Increased work of breathing with accessory muscle use.

Abdomen: Scaphoid, non-tender, non-distended. No hepatosplenomegaly. Normal bowel sounds.

Extremities: 1+ bilateral lower extremity edema. No clubbing or cyanosis.

Skin: Pale, poor skin turgor. No lesions.

Neurological: Alert and oriented x3. Cranial nerves II-XII intact. Motor strength 4/5 throughout, limited by fatigue. Sensation decreased to light touch in stocking distribution bilaterally.

ECOG Performance Status: 3 (deteriorated from 2 over past month)

**6. Hospital Course Summary:**

Mr. Williams was admitted for management of severe dyspnea and hypoxemia associated with progressive malignant pleural effusion and underlying advanced NSCLC. The patient had exhausted multiple lines of therapy, including immunotherapy, targeted therapy (sotorasib for KRAS G12C mutation), chemotherapy, and clinical trial participation.

On admission, therapeutic thoracentesis removed 1,500 mL of serosanguineous fluid with temporary symptomatic improvement. Pleural fluid cytology confirmed persistent malignant disease. Drainage through existing tunneled pleural catheter was optimized with daily drainage of 250-500 mL.

Despite fluid removal, the patient's respiratory status continued to deteriorate with progressive hypoxemia requiring escalating oxygen support to 6L via nasal cannula. CT chest performed on hospital day 5 showed progression of pleural disease, increasing parenchymal involvement, and lymphangitic spread. Pulmonary function was severely compromised with an estimated FEV1 <30% predicted.

Palliative care was consulted for symptom management, and extensive goals of care discussions were conducted with the patient and family. The patient expressed a desire to focus on comfort measures and quality of life, declining intubation or mechanical ventilation.

A comprehensive multi-disciplinary meeting determined that further anti-cancer therapies would not provide clinical benefit given the patient's deteriorating performance status and history of progression on multiple prior therapies.

Symptom management was optimized with around-the-clock long-acting opioids, breakthrough medication for dyspnea and pain, low-dose benzodiazepines for anxiety, and non-pharmacological interventions. Steroids were initiated for dyspnea palliation.

Patient died on 2025-04-14 with good symptom control.

**9. Lab Values (Excerpt):**

| **Parameter** | **Baseline (3/2021)** | **Previous Visit (2/2025)** | **Admission (4/3/2025)** | **Discharge (4/14/2025)** | **Units** | **Reference Range** |
| --- | --- | --- | --- | --- | --- | --- |
| WBC | 8.2 | 10.8 | 12.5 | 13.2 | × 10^9/L | 4.0-11.0 |
| Hemoglobin | 13.8 | 10.2 | 9.4 | 9.2 | g/dL | 13.5-17.5 (M) |
| Hematocrit | 41.4 | 30.6 | 28.2 | 27.6 | % | 41.0-53.0 (M) |
| Platelets | 285 | 256 | 268 | 274 | × 10^9/L | 150-400 |
| Creatinine | 1.1 | 1.5 | 1.6 | 1.7 | mg/dL | 0.7-1.3 |
| eGFR | 68 | 46 | 43 | 40 | mL/min | >60 |
| BUN | 18 | 28 | 32 | 36 | mg/dL | 7-20 |
| Sodium | 138 | 134 | 132 | 133 | mmol/L | 135-145 |
| Potassium | 4.2 | 4.4 | 4.6 | 4.5 | mmol/L | 3.5-5.0 |
| Chloride | 102 | 96 | 94 | 95 | mmol/L | 98-107 |
| Bicarbonate | 25 | 30 | 32 | 31 | mmol/L | 22-29 |
| Glucose | 146 | 164 | 172 | 156 | mg/dL | 70-100 |
| Albumin | 3.8 | 3.1 | 2.8 | 2.7 | g/dL | 3.5-5.0 |
| Total Protein | 7.0 | 6.2 | 5.8 | 5.7 | g/dL | 6.4-8.2 |
| LDH | 248 | 365 | 412 | 425 | U/L | 125-220 |
| TSH | 4.2 | 2.8 | 3.1 | - | mIU/L | 0.4-4.0 |

**Pleural Fluid Analysis (4/3/2025):**

- Appearance: Serosanguineous
- RBC: 15,200/mm³
- WBC: 1,250/mm³ (predominantly lymphocytes)
- Protein: 4.8 g/dL
- LDH: 385 U/L
- Glucose: 58 mg/dL
- pH: 7.26
- Cytology: Positive for malignant cells consistent with adenocarcinoma

**Arterial Blood Gas (4/3/2025) on 4L O₂:**

- pH: 7.36
- pCO2: 48 mmHg
- pO2: 68 mmHg
- HCO3: 31 mEq/L
- O2 Saturation: 92%

**Electronically Signed By:**
Dr. M. Johnson (Medical Oncology) Date/Time: 2025-04-14 16:45

Dr. L. Garcia (Pulmonology) Date/Time: 2025-04-14 14:30

Dr. V. Sharma (Palliative Care) Date/Time: 2025-04-14 15:20
